# Electronic and Magnetic Properties of Ferrous Iron in a True Square‐Planar Molecular Environment

**DOI:** 10.1002/chem.202501474

**Published:** 2025-06-18

**Authors:** Tim Marcel Diederich, Tim Wehland, Maximilian Schrodt, Nikolai Kochetov, Alexander Schnegg, Carlos M. Jimenez‐Muñoz, Vera Krewald, Lingmei Ni, Nicole Segura Salas, Ulrike I. Kramm, Joachim Ballmann, Markus Enders

**Affiliations:** ^1^ Institute of Inorganic Chemistry Heidelberg University Im Neuenheimer Feld 270 69120 Heidelberg Germany; ^2^ Max Planck Institute for Chemical Energy Conversion 45470 Mülheim an der Ruhr Germany; ^3^ Department of Chemistry Quantum Chemistry TU Darmstadt Peter‐Grünberg‐Str. 4 64287 Darmstadt Germany; ^4^ Eduard‐Zintl‐Institute Catalysts and Electrocatalysts group, TU Darmstadt Otto‐Berndt‐Str. 3 64287 Darmstadt Germany

**Keywords:** molecular magnetism, iron, phthalocyanines, electronic structure

## Abstract

The electronic and magnetic properties of ferrous iron in the iron(II)‐2,3,9,10,16,17,23,24‐octakis(2,6‐diisopropylphenoxy)phthalocyanine (FePc^OAr^), exhibiting a true square‐planar molecular environment, are investigated. Inhibition of intermolecular interactions by steric substituents allows detailed investigation of the electronic structure arising from the planar geometry of the d^6^ electron configuration. Complementary magnetometry, Mössbauer, FD‐FT THz‐EPR (frequency‐domain Fourier‐transform terahertz electron paramagnetic resonance) and pNMR (nuclear magnetic resonance of paramagnetic molecules) spectroscopies show that FePc^OAr^ has an *S *= 1 ground state with large positive axial zero‐field splitting (ZFS) and a strongly anisotropic *g*‐tensor, with two *g*‐values much larger than the free electron *g*‐value and one smaller. Correlation between the magnetic properties and the electronic structure is provided by high‐level quantum chemical calculations. The calculations indicate a nearly triply degenerate ground level, in which spin‐orbit coupling mixes the isolated ^3^A_2g_ ground state with two excited ^3^E_g_ states, whose energy gaps to the ground state are almost identical. These findings provide valuable insights in the electronic structure of iron phthalocyanines and the long‐standing discussion on their true electronic ground level, which has important implications for the application of this important class of complexes in catalysis and magnetic materials.

## Graphical abstract

Spin Hamiltonian parameters of an iron(II) phthalocyanine are obtained by pNMR, THz‐EPR, and quantum chemical calculations. Large zero field splitting and the peculiar *g*‐tensor pattern originate from mixing of the ^3^A_2g_ ground state with two ^3^E_g_‐excited states of almost the same energy. This forms the basis for correlations between magnetism, structure, and reactivity of iron in this important coordination environment.

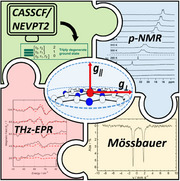

## 1 Introduction

Iron in a square planar coordination environment possesses extraordinary electronic properties that manifest in a rich reactivity. As an example, the axial binding sites are of intermediate strength so that substrates can be bound and liberated (e.g., O_2_ transport in hemoglobin).^[^ ^1^ ^]^ On the other hand, oxygen can be reduced at square‐planar iron centers, a reaction which is essential in aerobic biological processes ^[^ ^2^ ^]^ and increasingly important in technical applications for a hydrogen economy.^[^ ^3^ ^]^ A strong focus of recent research lies in the substitution of noble metals by base metals like iron, for instance replacing platinum catalysts in proton exchange membrane fuel cells.^[^ ^4^ ^]^ For the oxygen reduction reaction, the so‐called iron‐nitrogen‐carbon (FeNC) catalysts are promising.^[^ ^5^ ^]^ They contain square planar iron ligated by four nitrogen atoms.^[^ ^6^, ^7^, ^8^ ^]^ While there is common agreement on this square planar configuration, it is still debated whether the nitrogen is of pyridinic or pyrrolic nature, if there is an additional axial ligand and to what extent functional groups or iron impurities affect the catalytic properties.^[^ ^9^, ^10^ ^]^ In contrast to FeNC catalysts, molecular systems like iron porphyrins or iron phthalocyanines are well defined and much easier to study. The strong interaction of the four nitrogen atoms with the iron atom leads to a destabilization of the d_x2‐y2_ orbitals so that high spin complexes are disfavored. On the other hand, the other d‐orbitals are of similar energy so that the electrons occupy four d‐orbitals resulting in two or three singly occupied orbitals (*S *= 1 for d^6^‐Fe^2+^ or *S *= 3/2 for d^5^‐Fe^3+^).^[^ ^11^, ^12^ ^]^ This special electronic situation together with the redox properties of both, the iron center and the ligand opens many options for substrate binding and transformation in a catalytic and stoichiometric manner.^[^ ^13^ ^]^ The electronic situation manifests itself in a large magnetic anisotropy originating from *g*‐anisotropy and/or zero‐field splitting (ZFS).^[^ ^14^, ^15^ ^]^ Unsubstituted iron(II) phthalocyanine (FePc) has an extremely low solubility due to π‐stacking leading to molecular aggregation and consequently most investigations have been performed on solid FePc samples. Intra‐ as well as intermolecular effects must be considered since the Fe‐Fe distance is relatively short in the solid state influencing for example the spectroscopic signature.^[^ ^16^ ^]^ Soluble FePc derivatives often have axial ligands coordinated changing the electronic structure dramatically and rendering the Fe centers hardly accessible by substrates.

FePc is paramagnetic in the solid state and the magnetic susceptibility at room temperature is 3.96*μ*_B_.^[^ ^14^, ^15^, ^17^ ^]^ The temperature dependence was fitted to an *S *= 1 Spin Hamiltonian (SH, eq. 1) with pronounced ZFS. Strong ferromagnetic Fe‐Fe couplings render FePc a molecular ferromagnet below 10 K.^[^ ^11^ ^]^ An unambiguous determination of the electronic and magnetic properties of iron(II) phthalocyanines should therefore be based on measurements in both solids and liquid solutions. While the spin ground state of metal phthalocyanines has been a topic of intense research with different experimental and quantum chemical approaches, recent studies on FePc confirm the triplet ground state.^[^ ^18^, ^19^, ^20^, ^21^, ^22^ ^]^ Solution magnetic studies of FePc do not exist due to its very low solubility. However, related iron porphyrin derivatives were analyzed by solution NMR spectroscopy in benzene or toluene as solvents.^[^ ^23^, ^24^ ^]^ The magnetic anisotropy of a partially deuterated iron porphyrin was extracted from residual quadrupolar couplings (*RQC*) in toluene solution.^[^ ^25^ ^]^ In these studies, the aromatic solvents were treated as noncoordinating molecules so that the data were attributed to molecules with four coordinate iron centers. However, we recently showed that a soluble iron(II) phthalocyanine coordinates aromatic solvent molecules leading to equilibria of 4, 5, and 6 coordinated iron centers.^[^ ^26^ ^]^

Although magnetic properties in phthalocyanines have been studied since several decades, the exact determination of their characteristic *g*‐ and ZFS‐values and their explanation in terms of their electronic structure are still a matter of debate. Problems in the experimental determination of *g*‐ and ZFS values in even‐spin systems arise from the fact that they are EPR silent in conventional electron paramagnetic resonance (EPR) spectrometers, when the ZFS exceeds the energy of the microwave quanta in the EPR spectrometer. Alternatively, applied superconducting quantum interference device (SQUID) magnetometry, however, has limitations with respect to the accurate and independent assignment of *g*‐ and ZFS values. Finally, large magnetic anisotropies arise from spin‐orbit coupling between the ground state and nearby excited states. However, magneto‐structural correlations in this class of systems require quantum chemical methods that have only recently become available with the necessary accuracy.

In the present work, we investigated the soluble, square planar coordinated d^6^‐iron(II) system FePc^OAr^ (**1**), in which intermolecular magnetic interactions are irrelevant so that the pure intramolecular properties can be analyzed. We chose a substituted iron(II) phthalocyanine as a model compound because it is easy to synthesize on a large scale. The substituents prevent close Fe‐Fe contacts even in the solid state and improve the solubility in organic solvents dramatically so that the properties of isolated molecular units can be analyzed in solution and in the solid state. Magnetometry with a SQUID in combination with field‐dependent FD‐FT THz‐EPR and Mössbauer spectroscopies were employed to assign the spin state and the electronic *g*‐ and ZFS‐values. Magnetic characterization at cryogenic temperatures is complemented by the determination of the temperature dependence of two paramagnetic NMR effects in the liquid state, namely residual quadrupolar coupling (RQC) and pseudo contact shift (PCS). Complete Active Space Self Consistent Field (CASSCF) calculations with subsequent *N*‐electron valence perturbation theory (NEVPT2) provide a full picture of the electronic structure and the origin of the experimentally determined *g*‐ and ZFS values from spin‐orbit coupling within the nearly triply degenerate electronic‐ground level. The latter was found to be formed by an isolated ^3^A_2g_ ground state with two excited ^3^E_g_ states, whose energy gaps to the ground state are almost identical.

## 2 Results

### 2.1 Solid‐State Properties of FePc^OAr^ (1)

The synthesis of **1** was reported recently, as well as its stoichiometric and catalytic reactions with molecular oxygen.^[^ ^26^ ^]^ Crystals of **1** could be obtained from a solution in hexamethyldisiloxane containing a small amount of benzene. The result of the x‐ray diffraction analysis is shown in Figure 2.^[^ ^27^ ^]^ Benzene molecules are incorporated in the lattice but do not interact with the iron centers. A sp^2^‐carbon atom of a diisopropylphenyl substituent from a neighbor molecule is 3.02 Å apart. The shortest Fe···Fe distance is 12.38 Å. These structural parameters allow the description of **1** as a fourfold coordinated square planar iron complex.

In Figure 3 the Mössbauer spectrum of **1** is given. The powder sample of **1** was stored under argon but the measurement itself was conducted in air at 298 K. The data were fitted assuming the presence of two doublets, named D1 and D2. For the main species D1 (92%), the isomer shift (IS) is 0.38 mm s⁻¹ and the quadrupole splitting (QS) is 2.65 mm s⁻¹. The isomer shift is identical to the value of β‐Fe^II^Pc, while the quadrupole splitting is slightly higher (β‐FePc: IS = 0.38 mm s⁻¹, QS = 2.58 mm s⁻¹).^[^ ^16^ ^]^ The QS in unsubstituted FePc is associated with a pseudo‐octahedral coordination, where the iron center interacts in axial direction with the aza nitrogen atoms of the neighboring metal complex. In **1** this coordination of the aza nitrogens is not possible, as shown in solid state molecular structure (see Figure 2). This indicates that the large QS of **1** has a different origin. The second species D2 is a minor compound (8%) and has Mössbauer parameters of IS = 0.22 mm s⁻¹ and QS = 0.92 mm s⁻¹. Such Mössbauer parameters can be assigned to ferric high‐spin sites (see Figure S7, Supporting Information).

Figure 4a depicts *χT* versus *T* curves of neat powder of **1** obtained with a SQUID magnetometer. The room temperature *χT* = 1.795 emu⋅K corresponds to *µ*_eff_  =  3.79*µ*_B_. Assuming pure spin magnetism $\mu_{\mathrm{eff}}=g_{e}\sqrt{S(S+1)}\mu_{B}$ and a total spin of *S* = 1 would give $\mu_{\mathrm{eff}}=2.83\mu_{B}$. Therefore, the larger measured *µ*_eff_ indicates *g*_iso_ > *g*_e_ = 2.00232. Lowering the temperature below ∼70 K leads to a sharp drop in *χT*, indicating substantial ZFS. A large positive axial ZFS and *g*_iso_‐values significantly larger than the *g*_e_ have been reported for related iron(II) phthalocyanines (see Table 1). SQUID magnetometry is a powerful method for the assignment of ZFS‐ and *g*‐values of integer spin states with large magnetic anisotropies. However, since the ZFS‐ and *g*‐values in simulations of *χT* versus *T* curves are highly correlated, it is often not possible to determine their anisotropy from simulation of SQUID data alone. As we have recently shown, complementary Frequency‐Domain Fourier Transform THz‐EPR (FD‐FT THz‐EPR) measurements are capable of determining ZFS‐ and *g*‐values independently.^[^ ^28^, ^29^ ^]^ Figure 4b depicts FD‐FT THz‐EPR spectra at *T* = 5 K, obtained with excitation energies from 40 to 90 cm^−1^ and at external magnetic fields ranging from 0 to 10 T. THz‐EPR Spectra are plotted as magnetic field division spectra (MDS), where raw spectra obtained at an external magnetic field *B*_0_ + 2 T are divided by a reference spectrum measured at *B*_0_. The THz‐EPR MDS at low magnetic field show a broad peak around 65 cm^−1^, which shifts, gains intensity and finally splits upon increasing the external magnetic field beyond 5 T. These peaks were assigned to EPR transitions from the *M_S_* = 0 to the *M_S_* = ± 1 levels. The splitting of the peak was assigned to a small rhombic component in the ZFS of **1** of *E* = 4.6 cm^−1^. Rhombic ZFS lifts the degeneracy of the *M_S_* = ± 1 levels at zero field by 2*E* (see Figure 4c for the corresponding spin‐energy level diagram) and leads to a corresponding splitting in the EPR spectrum. SQUID and THz‐EPR traces could be simulated using the Matlab^TM^ toolbox EasySpin^[^ ^30^, ^31^ ^]^ employing the following Spin Hamiltonian (SH):

(1) $$\hat{H}=D\left[{\hat{S}}_{z}^{2}-\frac{1}{3}S\left(S+1\right)+\frac{E}{D}\left({\hat{S}}_{x}^{2}-{\hat{S}}_{y}^{2}\right)\right]+\mu_{B}\;B_{0}\;g\;\hat{S}$$

**Table 1:** ZFS‐ and *g*‐values obtained from experimental data of complex **1** alongside values reported in literature on related compounds. Numbers in brackets are the uncertainties on the last digits of the given values. The ZFS parameter derived from *PCS* measurements is too large and therefore printed in grey.

Compound	Method	D/cm^−1^ and E/cm^−1^	*g*‐values	*µ*_eff_ / *µ*_B_	Ref.
**1**	*Magnetometry* *χT* vs *T*	*D *= 65 (5), *E* = 3.25 (3)	$\;g_{∥}$= 1.95(40), $\;g_{⊥}$= 2.82(40) $\;g_{\mathrm{avg}}$= 2.56 (20) $\;g_{\mathrm{iso}}$= 2.53 (20)	3.79	This work
**1**	THz‐EPR	*D *= 60.2 (2.0); *E* = 4.6 (2.0)	$\;g_{∥}$= 1.36(20), $\;g_{⊥}$= 3.03(40) $\;g_{\mathrm{avg}}$= 2.60 (20) $\;g_{\mathrm{iso}}$= 2.47 (20)		This work
**1‐D_8_**	NMR (*RQC*)	*D *= 80 (15); *E* = 0	$\;g_{∥}$= 1.83 (10), $\;g_{⊥}$= 3.10 (5) $\;g_{\mathrm{avg}}$= 2.67 (5) $\;g_{\mathrm{iso}}$= 2.74 (5)	3.7 (Evans Method)^[^ ^32^ ^]^	This work
**1**	NMR (*PCS*)	*D *= 165 (20); *E* = 0	$\;g_{∥}$= 2.31 (10), $\;g_{⊥}$= 3.0 (5) $\;g_{\mathrm{avg}}$= 2.79 (5)		
FePc	CASSCF/NEVPT2	*D *= 91.0	$\;g_{∥}=g_{\mathrm{zz}}$= 1.773 $\;g_{\mathrm{xx}}$= 3.231 $\;g_{\mathrm{yy}}$= 3.217, $\;g_{\mathrm{iso}}$= 2.740 $\;g_{\mathrm{avg}}$= 2.824		This work
β‐FePc	*Magnetometry*	*D* = 69.9; *E* = 0;	$\;g_{∥}$ = 1.93, $\;g_{⊥}$= 2.86 $\;g_{\mathrm{avg}}$ = 2.6 $\;g_{\mathrm{iso}}$ = 2.55	3.71	Ref. [14]
β‐FePc	*Magnetometry*	*D *= 64	$\;g_{\mathrm{avg}}$= 2.74	3.89	Ref. [15]
β‐FePc	*Magnetometry*			3.85	Ref. [33]
β‐FePc	*Magnetometry*			3.96	Ref. [17]
β‐FePc	*Magnetometry*	*D* = 62.4	$g_{\mathrm{avg}}$ = 2.7 (2)		Ref. [34]
α‐FePc	*Magnetometry*	*D* = 37	$g_{\mathrm{avg}}$ = 2.54		Ref. [11]

As can be seen in Table 1, across all methods, we find a large positive and axial ZFS and a ***g***‐tensor anisotropy with two *g*‐values that are substantially larger than the free electron *g*‐value ($g_{\mathrm{xx}}\approx \;g_{\mathrm{yy}}\;\left(=g_{⊥}\;\right)>g_{e}=2.0023$) and one *g*‐value smaller than $g_{e}$ ($g_{\mathrm{zz}}=g_{∥}\;<\;g_{e}$).

Here, the first term describes the axial and rhombic 2^nd^‐order ZFS with parameters *D* and *E*, respectively. The second term denotes the electron‐spin Zeeman term involving the *g*‐tensor. *g*_z_ is assumed as collinear to *D*_z_. With this model the ZFS‐ and *g*‐values summarized in Table 1 were obtained.

### 2.2 Solution Properties, NMR‐Analysis

**Figure 1:**
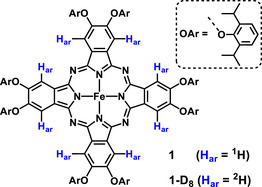
Iron compound **1** used in this study. The β‐H atom of the phthalocyanine is shown in blue.

Variable temperature (VT) NMR measurements of **1** were performed in methylcyclohexane‐D_14_ (Figure 5) and in toluene‐D_8_. In aromatic solvents different species are in equilibrium resulting in a non‐typical temperature behavior (Figure S2, S3, Supporting Information). Usually pNMR shifts decrease at higher temperature but the shifts of **1** increase when going from room temperature to higher temperatures. We attribute this behavior to temperature dependent equilibria of the solvent coordination to the iron center. Therefore, we used methylcyclohexane‐D_14_ as a solvent for a detailed analysis of the temperature dependence of the paramagnetic shift of **1** and isopropylcyclohexane for NMR shift and *RQC* effects of **1‐D_8_** (see Figure 1), respectively. These solvents were chosen as they hardly interact with the four‐coordinate iron center and allow NMR measurements in a temperature range from 147 K – 374 K (methylcyclohexane) and 184 K – 428 K (isopropylhyclohexane). The signal of H_ar_ (see Figure 1) was used as it gives the largest effect and the position relative to the paramagnetic center is well defined.

**Figure 2:**
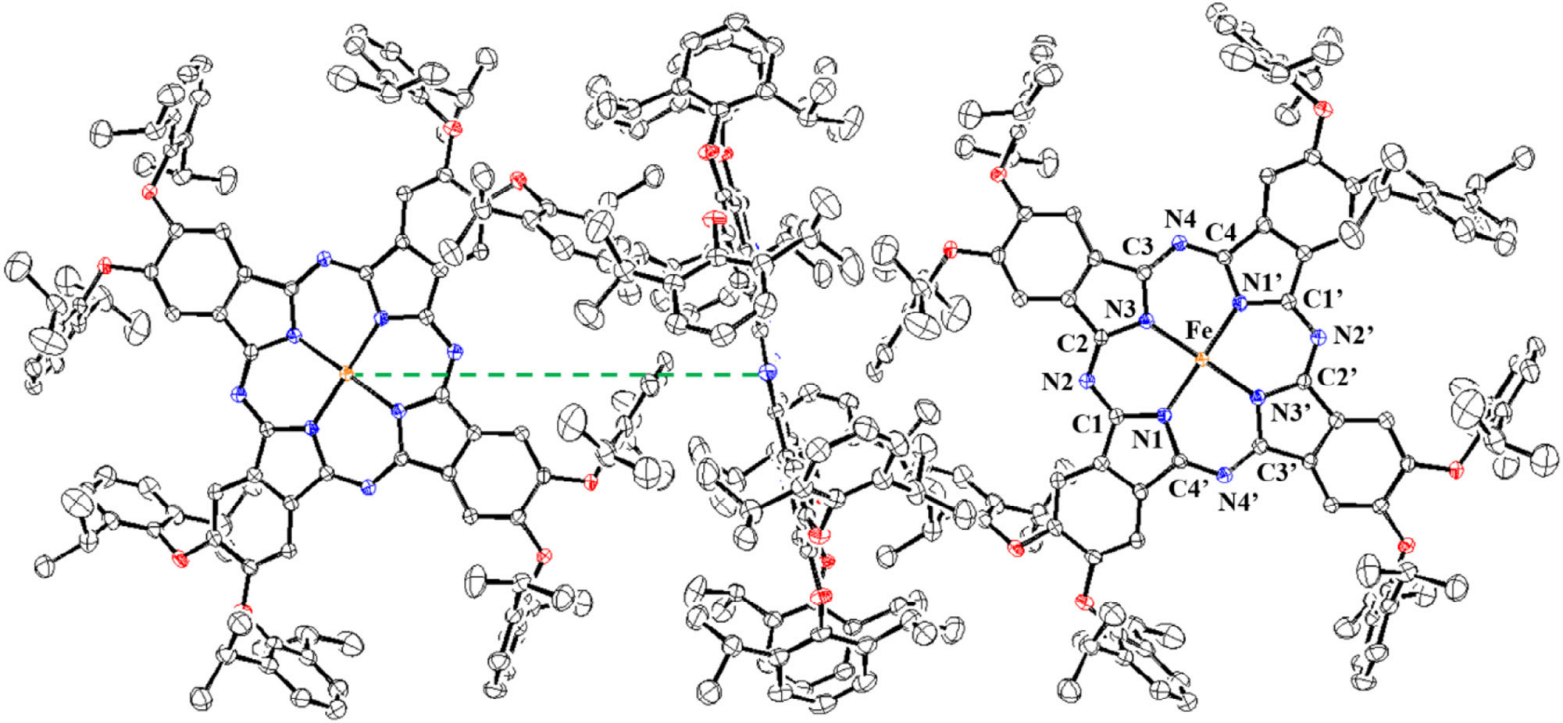
ORTEP‐depiction of solid state molecular structure of **1**. Hydrogens and solvent molecules (benzene) are omitted for clarity. Thermal ellipsoids are drawn at the 50% probability level. The shortest Fe‐Fe distance of 12.38474(8) Å is indicated by a green dashed line. Selected distances [Å] and angles (°): Fe‐N1 1.9350(12), Fe‐N3 1.9311(12), N1‐C1 1.3822(18), C1‐N2 1.3262(19), N2‐C2 1.3301(19), C2‐N3 1.3790(18), N3‐C3 1.3789(18), C3‐N4 1.3208(19), N4‐C4 1.3207(19), C4‐N1’ 1.3768(18), N1‐Fe‐N3 89.26(5), N3‐Fe‐N1’ 90.74(5), N1‐C1‐N2 127.00(13), C1‐N2‐C2 122.07(13), N2‐C2‐N3 127.24(13), C2‐N3‐C3 107.27(11), N3‐C3‐N4 127.95(13), C3‐N4‐C4 122.23(13), N4 C4 N11 127.86(13).

**Figure 3:**
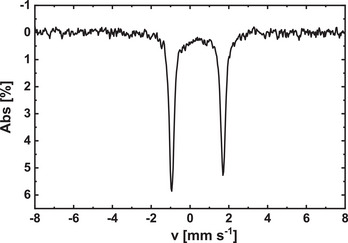
Mössbauer spectrum of the iron compound **1** obtained at 298 K (IS = 0.38 mm s⁻¹, QS  =  2.65 mm s⁻¹). IS is given relative to α‐Fe.

**Figure 4:**
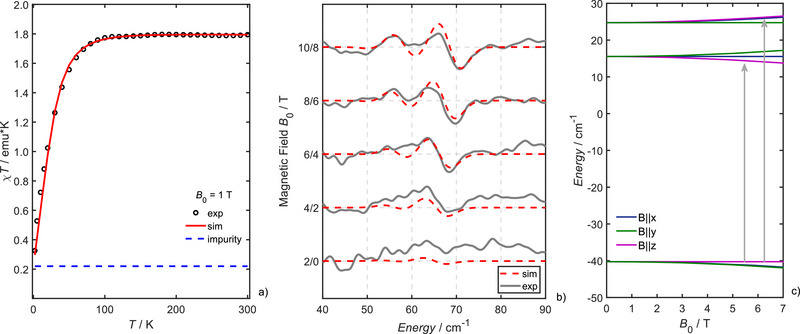
Magnetic characterization of **1**, a) *χT* versus *T* obtained at 1 T, (the blue dashed line indicates the contribution of 5% Fe(III), *S *= 5/2 impurities), b) FD‐FT THz‐EPR MDS at *T* = 5 K. Simulations (red dashed lines) obtained with Eq. 1 and parameters given in the text and Table 1 are plotted alongside experimental data (dots for *χT*, solid grey lines for FD‐FT THz‐EPR spectra). c) Calculated *S* = 1 spin‐energy levels versus external magnetic field calculated with the same set of spin Hamiltonian parameters as for the THz‐EPR simulations. Color code of the spin energy levels: $B_{0}$ aligned along the x‐ (blue), y‐ (green) and z‐axes (magenta) of the ZFS‐tensor. Vertical arrows indicate allowed EPR‐transitions for the z‐axis projection.

**Figure 5:**
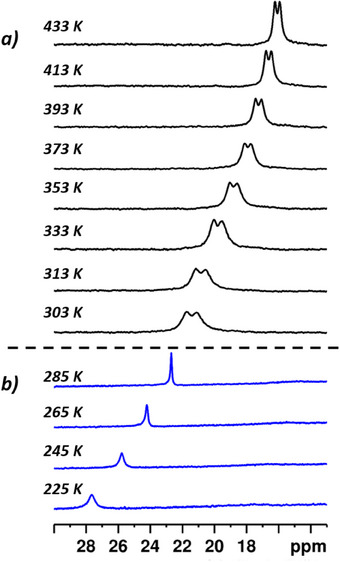
Excerpt from variable temperature ^2^H NMR spectra of **1** **‐** **D**_**8**_ in isopropylcyclohexane a) and ^1^H NMR spectra of **1** in methylcyclohexane‐D_14_ b). The spectra show the region of the H_ar_ signals.

We analyze the pNMR shift of H_ar_ by considering the different contributions to the experimental NMR shift, namely the orbital shift (*δ*_orb_), the pseudo contact shift (*PCS*), and the Fermi‐contact shift (*FCS*):

(2) $$\delta_{\exp}=\delta_{\mathrm{orb}}+PCS+FCS$$

The orbital shift is taken from the corresponding Zn‐complex using the same ligand (see Figure S4, Supporting Information). DFT calculation of the Fermi‐contact shift gives a value of zero for H_ar_ (see Supporting Information). Therefore, the paramagnetic ^1^H NMR shift can be interpreted by considering the *PCS* only. In an axial system the point‐dipole approximation gives a linear correlation between *PCS* and the magnetic anisotropy originating from the paramagnetism of the unpaired electrons located at the metal center (*Δχ*_ax,pd_) ^[^ ^35^ ^]^ as shown in by eq. 3.

(3) $$PCS=\frac{1}{12\pi r^{3}}\;\cdot \left[\Delta \chi_{\mathrm{ax},\mathrm{pd}}\left(3\mathrm{co}s^{2}\theta -1\right)\right]$$

Eq. 3 employs the point‐dipole approximation, where *r* is the length of the vector between the NMR nucleus and the paramagnetic center and *θ* is the angle between that vector and the magnetic axis. The total magnetic anisotropy of a molecule has several components, where the paramagnetic contribution is usually dominating. This anisotropy leads to a partial alignment of the dissolved molecules in the presence of the magnetic field of the NMR spectrometer. Consequently, NMR signals of quadrupolar nuclei are split to a multiplet with a multiplicity of 2*I* (*I* is the nuclear spin quantum number) and this effect is called residual quadrupolar coupling (*RQC*). Hence, ^2^H NMR signals are split to doublets if the molecule is partially oriented and the size of this splitting is proportional to the magnetic anisotropy and the square of the external magnetic field as shown in equation 4. The nonflexibility of the α‐H atom and the orientation of the C─H bond in the plane spanned by the phthalocyanine makes it a good sensor for both PCS and RQC analysis. Consequently, we prepared the deuterated analogue **1‐D_8_** (Figure 1) for studying the size and temperature dependence of the *RQC*. Similar to the *PCS*, the *RQC* depends on the magnetic anisotropy as shown in

(4) $$RQC=\frac{\left(e^{2}qQ/h\right)B_{0}^{2}}{20\mu_{0}kT}\;\cdot \left|\left[\Delta \chi_{\mathrm{ax}}\left(3\mathrm{co}s^{2}\vartheta -1\right)\right]\right|.$$

***B*_0_** is the strength of the magnetic field and ϑ is the angle between the ^2^H─C bond and the magnetic axis. In our case both, *θ* and ϑ have the same value of 90°. Here the magnetic anisotropy of the whole molecule has to be considered. In our case the eight phenoxy substituents are oriented orthogonal to the phthalocyanine so that the magnetic anisotropies in the ligand framework cancel to a large extent (see Supporting Information for details).^[^ ^36^ ^]^ Therefore, the total magnetic anisotropy originates almost exclusively from the paramagnetic contribution of the unpaired electrons located at the metal center. *RQC* measurements of a partially deuterated iron porphyrin using toluene as a solvent gave Δ*χ*_ax_ = ‐18.1 × 10^−32^ m^3^ at 298 K. ^[^ ^37^ ^] [^ ^25^ ^]^ However, toluene may act as a ligand so that the *RQC* data may come from a four‐, five‐ or six‐coordinated complex. For **1** we have shown that such equilibria exist (see above). We used **1‐D_8_** which dissolves in saturated hydrocarbons and measured *RQC*s at variable temperatures in isopropylcyclohexane as solvent. At 298 K we determined Δ*χ*_ax_ = –13.0 × 10^−32^ m^3^. The temperature dependence of Δ*χ*_ax_ calculated from *PCS* and *RQC* by eq. 2 and 3, respectively was fitted to eq. 5 which is a variant of an equation derived by Kurland and McGarvey for d‐block complexes in the presence of ZFS.^[^ ^38^ ^]^

(5) $$\begin{array}{c} \Delta \chi_{\mathrm{ax}}=\frac{\mu_{0}\mu_{B}^{2}S\left(S+1\right)}{3k}\cdot \left[\frac{\left(g_{∥}^{2}-g_{⊥}^{2}\right)}{T}-\frac{D}{T^{2}}\cdot \frac{\left(2S-1\right)\left(2S+3\right)\left(g_{∥}^{2}+\frac{1}{2}g_{⊥}^{2}\right)}{15k}\right] \end{array}$$

**Figure 6:**
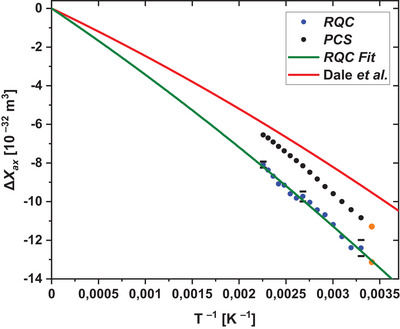
Fitting of Δ*χ_ax_* from the experimental *PCS* and *RQC* data at 16.45 T (700 MHz ^1^H) using eq 5. The data points from measurement at 22.32 T (950 MHz ^1^H) are marked as orange dots. Three black error bars are given for the *RQC* data. The red curve represents the anisotropy obtained by using data from Dale et al.^[^ ^14^ ^]^ SH parameters for the fit of the *RQC* data were selected as follows: *g*_||_ = 1.83, *g*_⊥_ = 3.10, *D* = 80 cm^−1^.

Eq. 5 nicely shows that the axial component of the magnetic anisotropy (Δ*χ*_ax_) has a temperature dependence of T^−1^ originating from *g*‐anisotropy and a temperature dependence of T^−2^ originating from ZFS. Therefore, both components can be accessed experimentally by measuring the temperature dependence of either PCS or RQC and the results are shown in Figure 6. Eq. 5 assumes the principal axis of the *g*‐tensor and the ZFS‐tensor to be collinear and it is a good approximation at higher temperatures where *D* « *kT*. The theoretical analysis confirmed that the main axes of the two tensors are aligned with the symmetry axes of the molecule (vide infra). *g*‐ and ZFS values from early literature data (Dale et al.^[^ ^14^ ^]^) predict a temperature behavior of Δ*χ*_ax_ which is not reproduced by our experimental data (red curve, Figure 6). Fitting our *RQC* data gives values which are very similar to the results from THz‐EPR of **1** and the calculations (see Table 1).

### 2.3 Discussion and Computational Analysis

The results from two NMR methods are different. Whereas the *RQC* method nicely reproduces the results obtained from THz‐EPR and calculations, the *PCS* method gives ZFS values that are too large. One possible reason for the poor *PCS* result is the use of the point‐dipole approximation which is not appropriate if unpaired electron spin is delocalized.^[^ ^39^ ^]^ Nevertheless, the determination of the sign of *D* is easily done by measuring *PCS* at different temperatures as the curvature of Δ*χ* versus *T* ^−1^ only depends on the sign of *D* (see eq. 5 and Figure 6).

The set of determined parameters is a clear indication of a degenerate ground level, in which the energetically lowest states^[^ ^40^ ^]^ have an energy gap comparable to the spin‐orbit coupling constant (SOC) constant of Fe(II) which was set here to ζ = 400 cm^−1^.^[^ ^41^ ^]^ As shown for the structurally related Fe(TPP)^[^ ^28^ ^]^ and recently extended to low‐coordinate complexes in general,^[^ ^29^ ^]^ the *g*‐ and ZFS‐anisotropy observed is in accordance with a triply‐degenerate ground level in which SOC spin mixes an energetically separated ground state with two higher‐lying states, for which the energy difference between the higher‐lying states is small compared to their energy difference to the ground state.^[^ ^28^ ^]^ As can be seen above, common rules derived from second order perturbation theory to assign *g*‐values in 3d metals, where an excitation from a doubly occupied to a singly occupied state leads to *g*‐values larger than *g*_e_ and excitation to an unoccupied orbital to a *g*‐value smaller than *g*_e_, is not generally applicable for degenerate ground levels.^[^ ^28^ ^]^

Experimental magnetochemistry alone cannot assign the states which are mixed in the ground level. The assignment of the electronic ground state structure of the triplet ground state phthalocyanines via magneto‐structural correlations has been the subject of an ongoing debate since Klemm's early susceptibility studies on phthalocyanines.^[^ ^17^ ^]^ Depending on the experimental method and the applied level of theory ^3^E_g_^[^ ^18^, ^42^ ^]^, ^3^A_2g_^[^ ^43^, ^44^ ^]^ or even ^3^B_2g_^[^ ^45^ ^]^ have been proposed as ground state. Furthermore, mixing of these states to a near‐degenerate ground level has been suggested.^[^ ^20^, ^46^ ^]^ To finally resolve this question and identify the ground level of the triplet state in FePc we are comparing our experimentally obtained parameters with state‐of‐the‐art wavefunction calculations.

**Figure 7:**
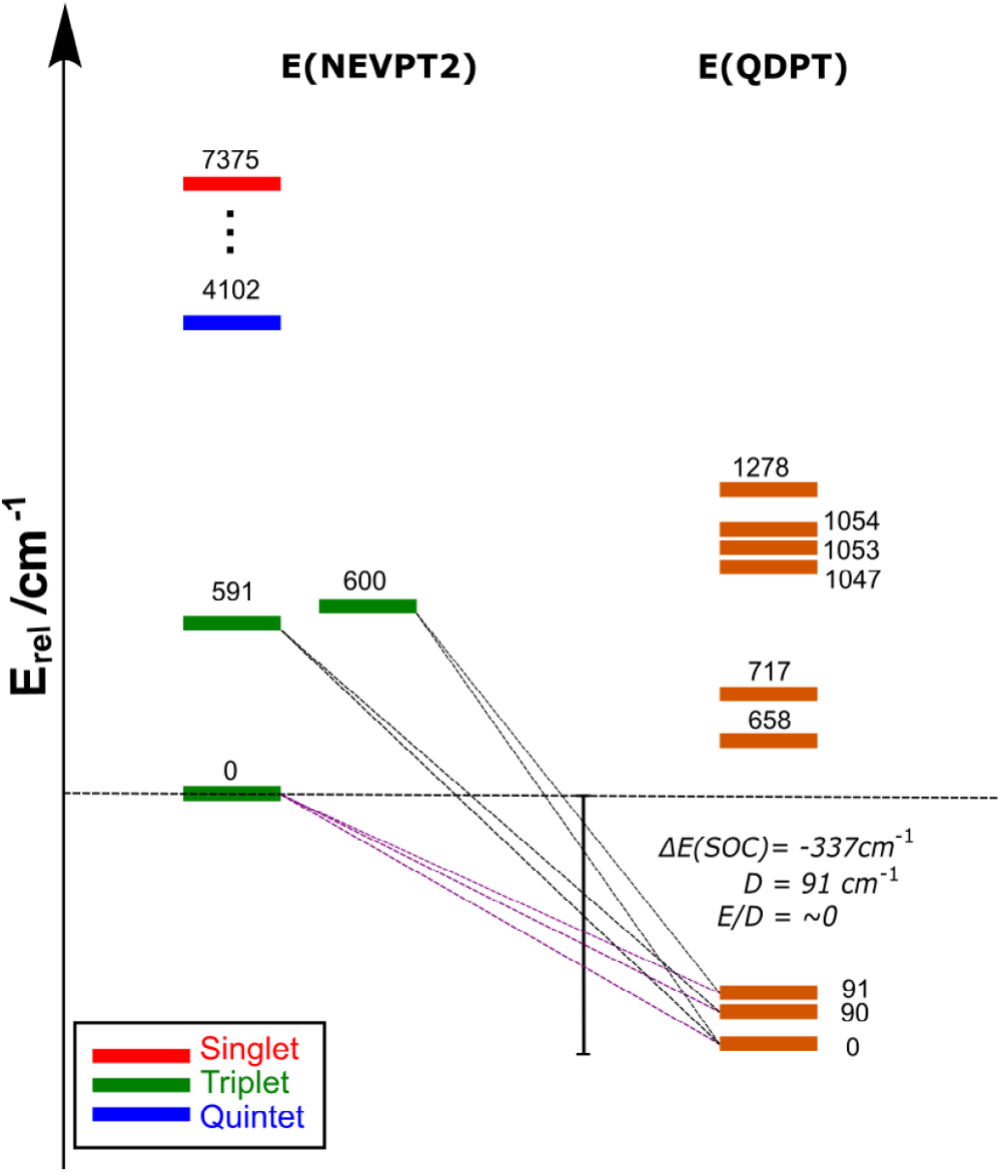
Representation of the CASSCF/NEVPT2 states (left) and SOC mixed states (right). The contribution of each low‐lying triplet (one ^3^A_2g_ and two ^3^E_g_) to each of the nine SOC‐states is shown in Table S12 (Supporting Information).

First, we compare the fully substituted complex **1** (FePc^OAr^) with the unsubstituted FePc at the DFT level of theory. Both structures were fully relaxed at the TPSS/def2‐TZVP:def2‐SVP level of theory (see Figure 7 and Computational Details). The ligand truncation has a very limited influence on the geometry as exemplified by the Fe‐N distances and angles in Figure S8 (Supporting Information). Using single‐point calculations (PBE0/def2‐TZVP:def2‐SVP), the triplet ground state is reproduced for the fully substituted and the truncated complexes. The lowest quintet is predicted at 7.3 kcal mol^−1^ ([FePc^OAr^]: 25.7 kcal mol^−1^) and the lowest singlet at 14.5 kcal mol^−1^ ([FePc^OAr^]: 32.5 kcal mol^−1^) above the triplet ground state. For the quantum chemical analysis, we used the fully DFT optimized structure of FePc. The reduction of the ligand complexity is necessary because we need to include enough ligand orbitals in the active space of our calculations (see below). Tarrago and coworkers ^[^ ^28^ ^]^ reported an almost triply degenerate electronic ground state for ferrous tetraphenylporphyrin and attributed the magnetic properties to this electronic structure. Given the similar coordination environment, we anticipated a similar multi‐reference behavior for the FePc complex, requiring complete active space calculations with inclusion of SOC. CASSCF/NEVPT2 calculations were carried out using an active space of 18 electrons and 15 orbitals, that is, (18,15) in common nomenclature. This active space included the five Fe 3d orbitals, five doubly occupied and five virtual ligand orbitals akin to Gouterman orbitals, see Figure S9 (Supporting Information). ^[^ ^28^, ^47^, ^48^, ^49^ ^]^

At the CASSCF/NEVPT2 level of theory, the electronic ground state is predicted to be ^3^A_2g_. The main configuration (81%) has an occupation pattern (d_xy_)^2^(d_xz_,_yz_)^2^(d_z_^2^)^2^; any remaining configurations have contributions of less than 4% weight. Two states of the type ^3^E_g_ are predicted to be at 591 cm^−1^ and 600 cm^−1^ above the ground state. These two states have very similar main configurations (71%) with an orbital occupation pattern of (d_xy_)^2^(d_xz_,_yz_)^3^(d_z_^2^)^1^ that differs only in the occupation of the d_xz_,_yz_ orbitals. In both ^3^E_g_ states, a common secondary configuration with an orbital occupation pattern of (d_xy_)^1^(d_xz_,_yz_)^3^(d_z_^2^)^2^ contributes approx. 10% of the total weight, while the remaining configurations have contributions of less than 4% weight. The three states (one ^3^A_2g_ and two ^3^E_g_) are well isolated from energetically higher lying states: the next lowest quintet and triplet are predicted at 4102 cm^−1^ and 5091 cm^−1^ above the ^3^A_2g_ ground state, respectively (see Table S10, Supporting Information).

The energy range of the three lowest CASSCF/NEVPT2 states is in the same order of magnitude as the effective SOC constant (*ζ*) for Fe(II), which strongly suggests that mixing via SOC will be relevant.^[^ ^41^ ^]^ Using the QDPT implementation for SOC effects (see Computational Details),^[^ ^50^, ^51^ ^]^ a group of nine SOC states results from the mixing of the triplet CASSCF/NEVPT2 roots corresponding to the ^3^A_2g_ and the ^3^E_g_ irreducible representations. As shown in Figure 7, at the SOC‐CASSCF/NEVPT2 level of theory, the lowest SOC state arises from the mixing of the *M*_s_ components of the three lowest triplets (CASSCF/NEVPT2 root 0, 1, and 2). Similarly, the second and third SOC states involve contributions from roots 0 and 1, and roots 0 and 2, respectively. These three SOC states share a dominant contribution from the root 0 with ∼76% resulting in a quasi‐triply degenerate ground state with energy separations of ∼90 cm^−1^ and ∼91 cm^−1^ respectively. The remaining six SOC states lie below 1300 cm^−1^ (<|10*ζ*|), also originate from the coupling between the same CASSCF/NEVPT2 triplets, and are energetically well separated from the next accessible state (SOC state 9 at ∼4378 cm^−1^), see Table S11 (Supporting Information). While the first three SOC states arise predominantly from the lower‐energy root (root 0), the remaining six SOC states involve stronger contributions from the higher‐energy triplet roots (roots 1 and 2). Nevertheless, all SOC states belong to the same spin–orbit coupled manifold formed by the interaction of the ^3^A_2g_ and the ^3^E_g_ irreducible representations under SOC.

Based on QDPT‐SOC data, the ZFS was predicted to be *D* = 91 cm^−1^. This value is similar to the energy separation between the SOC ground state and the second lowest‐lying SOC state.^[^ ^52^ ^]^ The *g*‐factor was predicted to be *g_iso_ *= 2.740 with the three components *g*_xx_ = 3.231, *g*_yy_ = 3.217, *g*_zz_ = 1.773 showing an axial configuration. Comparing these results with the previously stated experimental measurements in Table 1, the isotropic value for the *g*‐factor is in good agreement with the reported values for complex **1** and corresponds to the signature of a quasi‐triply degenerate ground state with two almost‐degenerate states out of the three. Regarding the ZFS, the predicted value is in the same order of magnitude as the experiment showing only a small deviation. Several CASSCF/NEVPT2 calculations were performed using the different spin–orbit coupling (SOC) flags available in ORCA to evaluate their effects on the computed *g*‐factor and ZFS‐values. The results of these calculations are summarized in Table S13 in the Supporting Information, showing that the resulting *g*‐tensors and ZFS values exhibit only a small variation across the different SOC settings. These results suggest that the computed magnetic properties are not strongly affected by the employed SOC schemes.

## 3 Conclusion

We present a comprehensive analysis of the electronic and magnetic properties of FePc^OAr^, a true square‐planar Fe(II) phthalocyanine complex. The enhanced solubility and structural stability of FePc^OAr^, as well as the isolation of its iron center from neighboring molecules, make it an ideal model system for studying intramolecular properties of this important molecular class. SQUID magnetometry in combination with Mössbauer and THz‐EPR spectroscopies confirms a triplet ground state with significant positive, axial ZFS and a peculiar *g*‐tensor anisotropy, with two *g*‐values much larger than *g*_e_ and one smaller value. Spin Hamiltonian parameters obtained at cryogenic temperatures are compared to values obtained by different pNMR approaches in the liquid state. Measurement of the pseudo contact shift in the NMR spectrum is the simplest and does not require any special equipment, but in the present case, yields too large ZFS values. In contrast, the measurement of the quadrupolar residual coupling in deuterated **1** gave results that were in a good agreement with their low temperature counterparts. The obtained Spin Hamiltonian parameters are important comparative values for the general understanding of magneto structural correlations in phthalocyanines and related low coordinate metal sites. Knowledge of the magneto‐structural correlations in low‐coordinate metal centers is particularly important when their atomic structure is not known and detailed quantum chemical calculations are not possible. This is the case, for example, in heterogeneous single atom catalysts, such as FeNC materials obtained by pyrolysis. In these cases, magneto‐structural correlations can be employed for conclusions about the electronic structure without the need for quantum chemical calculations. Equally important, the experimentally determined ZFS‐ and *g*‐tensor anisotropies benchmark quantum chemical calculations, providing clear assignment of the electronic structure. CASSCF calculations revealed a nearly triply‐degenerate ground level that is formed by an isolated ^3^A_2g_‐ground state with two ^3^E_g_‐excited states, whose energy gaps to the ground state are nearly identical. We find that the large ZFS and the peculiar *g*‐tensor pattern originate from in‐state and out‐of‐state SOC mixing of the ^3^A_2g_ and ^3^E_g_‐states, similar to other square‐planar complexes. This assignment clarifies a longstanding discussion on the character of the electronic ground level in phthalocyanins and provides the basis for a thorough understanding of the catalytic properties of this important class of molecules. Considering that our calculations focus on the isolated molecule, previous reports have shown that minor perturbations such as surface adsorption, peripheral substitutions or axial ligation can shift the balance between low‐lying triplet states in macrocyclic ligands.^[^ ^28^, ^53^, ^54^ ^]^ The sensitivity of the Fe(II) electronic structure to external conditions underscores the need to interpret magnetic properties in the context of specific experimental environments.

## 4 Experimental Section

**Synthetic procedures and analytical data** The synthesis of **1** was published recently ^[^ ^26^ ^]^ but without single‐crystal X‐ray analysis. Crystals of **1** have been obtained from a solution in hexamethyldisiloxane (HMDSO) and traces of benzene by slow conversion of an amorphous solid to single crystals. As **1** reacts quickly with oxygen, the whole procedure was performed in the inert atmosphere of a glove box. The deuterated compound was synthesized by procedures published for the non‐deuterated derivative: 1,2‐dichloro‐4,5‐diiodobenzene‐3,6‐d_2_ was prepared by iodination of 1,2‐dichlorobenzene‐D_4_^[^ ^55^ ^]^ and was subsequently converted to 4,5‐dichlorophthalonitrile‐3,6‐D_2_ by Pd‐catalysed cyanation with Zn(CN)_2_.^[^ ^56^ ^]^ Reaction with 2,6‐diisopropylphenol using K_2_CO_3_ as a base leads to 4,5‐bis(2,6‐diisopropylphenoxy) phthalonitrile‐3,6‐D_2_^[^ ^57^ ^]^. The hexacoordinated iron(II)phthalocyanine **1**‐**D_8_** (tBuNC)_2_ was prepared and purified by a procedure published by Bezzu et al.^[^ ^58^ ^]^ and four‐coordinate **1‐D_8_** by heating the latter compound to 250 °C for 3 hours in a vacuum.^[^ ^26^ ^]^

^2^H‐NMR of **1‐D_8_** (Isopropylcyclohexane, 700 MHz, 303 K): 21.94 ppm (d, *RQC* = 59.4 Hz)

**NMR Measurements**: NMR samples were prepared in an argon atmosphere glovebox (*MB UNILAB from M. Braun Inertgas‐Systeme GmbH)* using thoroughly dried and deoxygenated solvents. The NMR tubes were flame sealed. NMR spectra were recorded on the NMR spectrometers *Bruker Avance II 400*, *Bruker Avance III 600*, *Bruker Avance Neo 700, and Bruker Avance III 950*. In ^1^H‐ and ^13^C‐spectra chemical shifts (δ) are listed in ppm (parts per million) relative to tetramethylsilane (δ = 0 ppm) using residual solvent peaks for referencing. The temperature was calibrated using 99.8% methanol‐D_4_ and 99% ethylenglycol‐D_6_. **1** dissolves well in a range of polar and nonpolar organic solvents like tetrahydrofuran (thf), dichloromethane, toluene, pentane, or methylcyclohexane. It is practically insoluble in H_2_O. The signals in all NMR spectra are shifted due to the paramagnetism of the compound. Large differences in the ^1^H NMR shifts are observed depending on the solvent used. Positive paramagnetic shifts are observed in noncoordinating solvents like n‐pentane or methylcyclohexane and negative shift in a solvent with good coordination ability like thf. The inversion of the sign of the paramagnetic shift in thf arises from a change in magnetic anisotropy when going from a square‐planar coordination environment in noncoordinating solvents to an octahedral environment in coordinating solvents.

**^57^Fe Mössbauer spectrum** was collected using a spectrometer equipped with a ^57^Co/Rh source in transmission mode. The measurement was carried out at 298 K, in a velocity ranges of ± 8.8 mm s^−1^. 333 mg of the sample were filled in a PTFE (polytetrafluoroethylene) holder with a height of 3 mm and a diameter of 1.5 cm, and then sealed with TESA tape. The velocity was calibrated using the sextet lines of α‐Fe. Data fitting was performed using the Recoil software ^[^ ^59^ ^]^.

**SQUID magnetometry** Magnetic susceptibility of **1** was measured from powder samples of solid material in the temperature range 2–300 K by using a SQUID with a field of 1 T (MPMS‐7, Quantum Design, calibrated with standard palladium reference sample, error < 2%). Variable‐field variable‐temperature (VFVT) magnetization measurements were done at 1, 4, and 7 T in the temperature range 2–260 K with the magnetization equidistantly sampled on a 1/T temperature scale.

**FD‐FT THz‐EPR spectroscopy** FD‐FT THz‐EPR was performed at the THz beamline of the synchrotron BESSY II, Helmholtz‐Zentrum Berlin, Germany.^[^ ^60^ ^]^ This spectrometer allows for THz‐EPR measurements from ∼ 0.1 to 200 THz (∼ 3–6000 cm^−1^) employing a fully evacuated quasi‐optical beam path, a high‐resolution FTIR spectrometer (IFS 125, Bruker), a superconducting high‐field optical magnet (Cryogenic Limited, *B*_0_ = +12 T – 12T) with variable temperature insert (*T* = 1.5 K–300 K) and liquid He cooled bolometer detectors. Herein, the internal source of the FTIR spectrometer (Hg arc lamp) was used as THz source in combination with a liquid He cooled (4.2 K) bolometer. A detailed description of the spectrometer can be found elsewhere.^[^ ^60^ ^]^ Samples were measured as pressed pellets (∼44 mg powder sample of **1** and ∼76 mg Polyethylene), SH simulations were performed with EasySpin and its extension for frequency‐domain EPR.^[^ ^31^ ^]^ Throughout the report FD‐FT THz‐EPR spectra are plotted as magnetic field devision spectra (MDS). MDS for different external magnetic fields B_0_ are obtained by dividing the raw spectrum measured at B_0_ by the spectrum measured at B_0_ + 2 T. Data is shown offset for B_0_ and rescaled with a global normalization factor. In MDS, EPR resonances appear as negative (EPR resonance at B_0_) and positive (EPR resonance at B_0_ + 2 T) deviations from 1.

### Computational Details

*Density Functional Theory*. For all the calculations in this work, the software package ORCA v5.0.4 was used.^[^ ^61^, ^62^, ^63^ ^]^ The spin states of this species were studied using a combined protocol of geometry optimized and single point calculations with the possible spin multiplicities singlet, triplet, quintet, and septet. A true minimum is confirmed by evaluating the Hessian matrix and ensuring that all its eigenvalues are positive. The structure was optimized at the TPSS level of theory including the London dispersion correction D4 proposed by Grimme.^[^ ^64^, ^65^ ^]^ For the basis set the Ahlrichs basis set group was employed; due to the size of the system the def2‐TZVP basis set was used for the Fe and N atoms while def2‐SVP basis set was used for the C and H atoms.^[^ ^66^ ^]^ To deal with convergence problems and sufficient SCF accuracy, Kolmar's Direct Inversion in Iterative Subspace (KDIIS) algorithm was used, as well as TightSCF and TightOPT keywords as the threshold for numerical accuracy. Additionally, the Split‐RI‐J approximation and the required auxiliary basis set def2/J was included. The single‐point energies were obtained at the OPBE theory level also including London dispersion correction D4.^[^ ^67^, ^68^ ^]^ In this case the basis set used to describe the Fe atom was CP(PPP) which has been proved to well describe magnetic properties in systems with similar coordination environment.^[^ ^69^ ^]^ For the rest of the atoms in the system the def2‐TZVP basis set was used. The KDIIS algorithm was included and the TightSCF was used. The RIJCOSX approximation and the required auxiliary basis set def2/J was included.^[^ ^70^ ^]^

*Complete Active Space SCF*. For the implementation of a multi‐reference methodology and the calculation of the magnetic properties, a state‐average Complete Active Space SCF (SA‐CASSCF) was performed as implemented ORCA v5.0.4. Here, scalar relativistic effects were included in the framework of the Douglas‐Kroll‐Hess Hamiltonian (DKH), in consequence, the basis set DKH‐def2‐QZVPP was used for the Fe atom, the DKH‐def2‐TZVP basis set was used for N and C atoms and the DKH‐def2‐SVP was used for H atoms. The option Autoaux was used to provide auxiliary basis sets as required by the setup, and for the SCF accuracy the option TightSCF was used. A total of 60 states were calculated from the set of active orbitals optimized through SA‐CASSCF for the multiplicities singlet, triplet, and quintet (20 states respectively) using the resolution of identity (RI) approximation. The strongly‐contracted version of NEVPT2 was used on the converged SA‐CASSCF wave function for the implementation of dynamic correlation. ^[^ ^71^, ^72^ ^]^ The calculation of the spin‐orbit coupling (SOC) was implemented through the quasi‐degenerate perturbation theory (QDPT) with the mean‐field/effective‐potential approximation, while the *g*‐matrix was calculated in terms of the effective Hamiltonian arising of the CASSCF/NEVPT2 diagonal energies including SOC through the QDPT.^[^ ^50^, ^51^, ^73^ ^]^ The active space consisted of 18 electrons distributed in 15 orbitals which were optimized using as initial guess TPSS/def2‐TZVP:def2‐SVP orbitals. The active space orbitals are the 3d orbitals (3d_xy_, 3d_xz_, 3d_yz_, 3d_x_^2^_‐y_^2^, 3d_z_^2^) and a set of π‐orbitals delocalized over the phthalocyanine ring, which include the Gouterman set.^[^ ^28^, ^47^, ^48^, ^49^ ^]^ The shape of all the active orbitals for the FePc complex are shown in the Supporting Information.

## Supporting Information

The authors have cited additional references within the Supporting Information.^[^ ^74^, ^75^, ^76^, ^77^, ^78^, ^79^, ^80^, ^81^, ^82^, ^83^, ^84^, ^85^, ^86^, ^87^, ^88^ ^]^

## Conflict of Interests

The authors declare no conflict of interest.

## Supporting information



Supporting Information

## Data Availability

The data that support the findings of this study are available in the supplementary material of this article. Spectroscopic and computational data are provided at: https://doi.org/10.48328/tudatalib‐1787
