# Supplementary material for: Electronic and Magnetic Properties of Ferrous Iron in a True Square‐Planar Molecular Environment
Source: Chemistry. 2025 Jun 18;31(39):e202501474. doi: 10.1002/chem.202501474 (PMC12258675; doi:10.1002/chem.202501474)
Supplement: Supplementary file 1 — Supporting Information [file CHEM-31-e202501474-s001.pdf]

Supporting Information

Electronic and Magnetic Properties of Ferrous Iron in a True Square-Planar Molecular Environment

Tim Marcel Diederich[1], Tim Wehland[1], Maximilian Schrodt[1], Nikolai, Kochetov,[2] Alexander Schnegg[2], Carlos M. Jimenez-Muñoz[3], Vera Krewald*[3], Lingmei Ni [4], Nicole, Segura Salas[4], Ulrike I. Kramm[4], Joachim Ballmann[1], and Markus Enders*[1]*

* Corresponding authors: krewald@chemie.tu-darmstadt.de; alexander.schnegg@cec.mpg.de; markus.enders@uni-heidelberg.de

1 Institute of Inorganic Chemistry, Heidelberg University, Im Neuenheimer Feld 270, 69120 Heidelberg, Germany

2 Max Planck Institute for Chemical Energy Conversion, 45470 Mülheim an der Ruhr, Germany

3 Department of Chemistry, Quantum Chemistry, TU Darmstadt, Peter-Grünberg-Str. 4, 64287 Darmstadt, Germany

4 Eduard-Zintl-Institute, Catalysts and Electrocatalysts group, TU Darmstadt, Otto-Berndt-Str. 3, 64287 Darmstadt, Germany

1. NMR 2

2. Single Crystal X-Ray Diffraction 7

3. Mössbauer (additional data) 11

4. Calculations 12

6. References 21

1. NMR

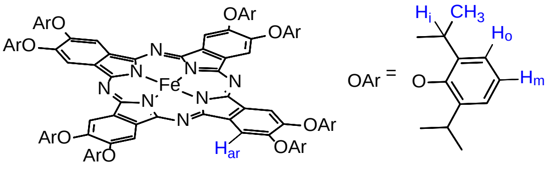

1

Figure S1. Designation of the ^{1}H NMR signals

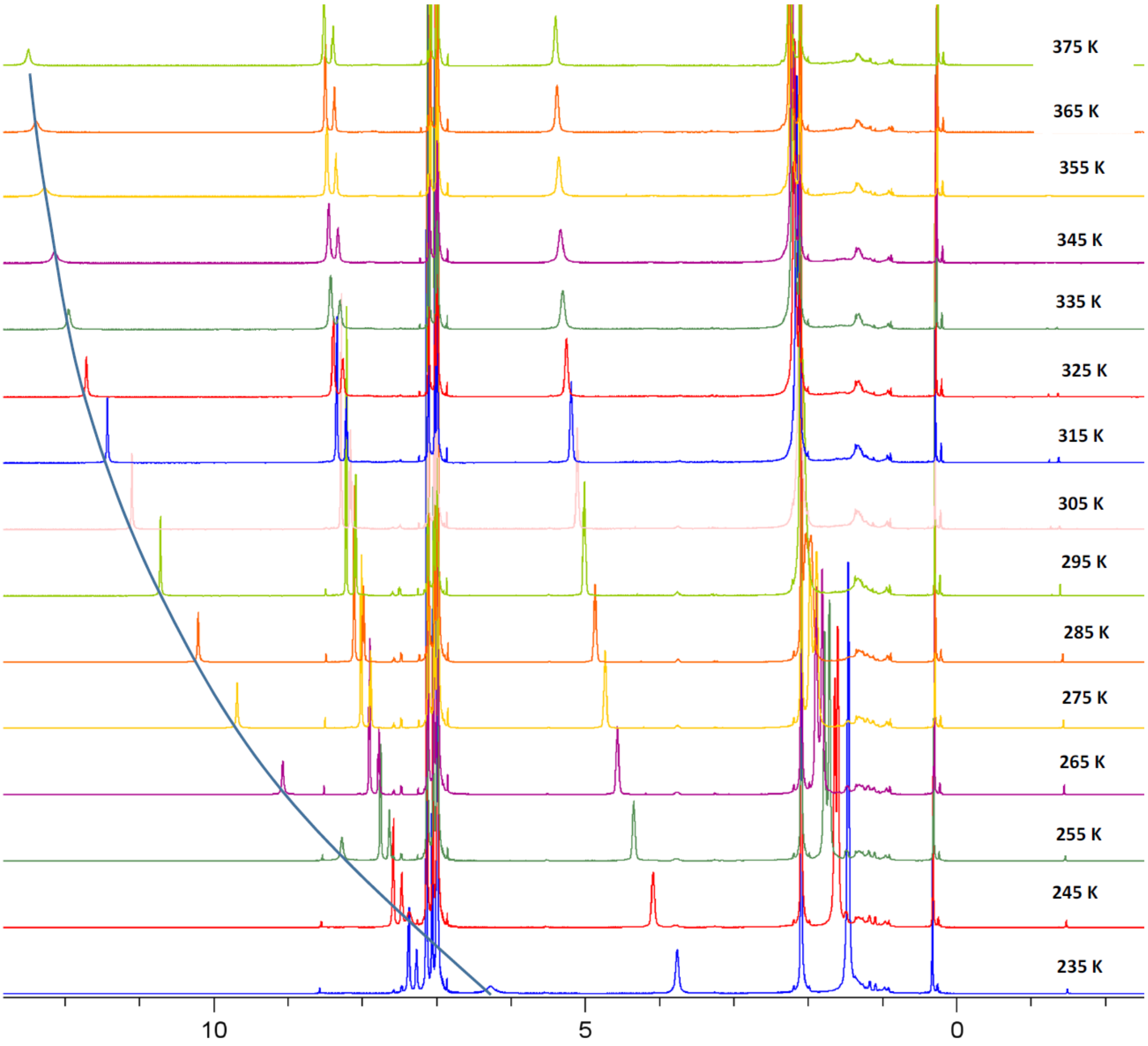

Figure S2. VT-NMR of **1** in toluene-D$_8$.

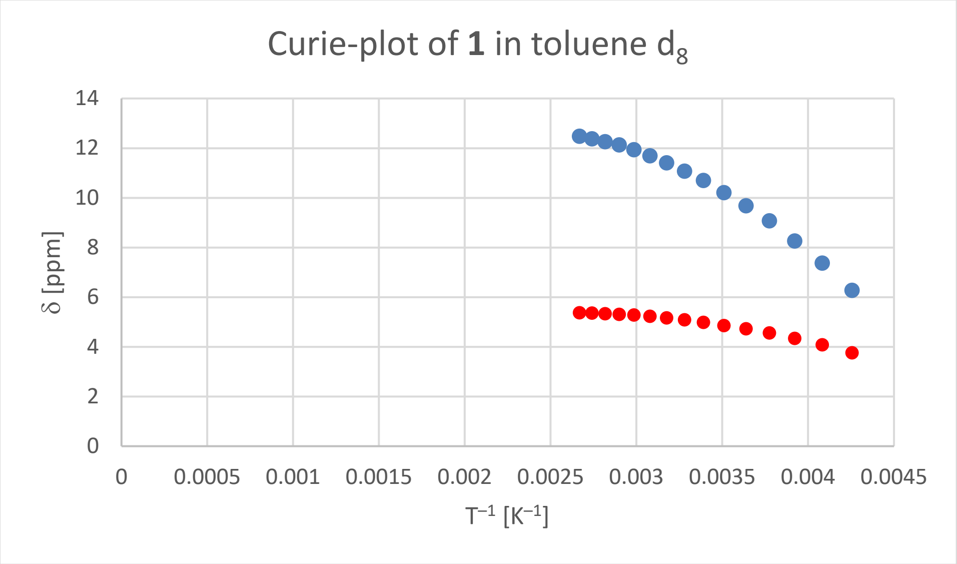

Figure S3: Curie-Plot of **1** in toluene-D_8. Plot of ^{1}H NMR shifts of signals of H_{ar} (blue) and H_i (red) versus $T^{-1}[K^{-1}]$.

Synthesis of H_2Pc^{OAr} (jl31)

4,5-bis(2,6-diisopropylphenoxy)phthalonitrile (1.5 g, 3.12 mmol, 1 eq.) is suspended in 10 mL of dry n-butanol under argon. Then 1,8-Diazabicyclo[5.4.0]undec-7-en (1.4 mL, 9.36 mmol, 3,0 eq.) is added and the reaction is stirred at 125 °C for 16 h. 150 mL of dichloromethane (DCM) is added to the dark green mixture and the organic phase is washed with water (2x400 mL). The solvent was removed in *vacuo* and the crude product is purified by flash column chromatography (SiO2/ DCM:PE 1:1). The product was obtained as a dark green powder (1.05 g, 545.6 µmol, 70 %).

^{1}H NMR (600.2 MHz, C_6D_6, 295K, jl31x/60010): δ[ppm]= 8.57 (s, 8H), 7.59 (t, J = 7.9 Hz, 8H), 7.50 (d, J= 7.9 Hz, 16H), 3.74 (sept, J= 6.9 Hz, 16H), 1.31 (broad-d, 96H), -0.84 (s, NH).

^{13}C NMR (150.9 MHz, C_6D_6, 295K, jl31x/60011): δ[ppm]= 151.2 (s, C_q), 149.1 (s, C_q), 141.7 (s, C_q), 131.0* (s, C_q), 126.4 (s, CH_{Ar}), 124.7 (s, CH_{Ar}), 107.4 (s, CH_{Ar}), 27.4 (s, CH), 24.5 (bs, CH_3), 22.9 (bs, CH_3), one C_q is missing, *determined by HMBC

Synthesis of $ZnPc^{OAr}$ (mm06)

H_2Pc^{OAr} (50.0 mg, 26.0 µmol, 1,00 eq.) was dissolved in pyridine (21.5 ml), anhydrous zinc acetate (47.7 mg, 26.0 µmol, 1.00 eq.) was added, and the mixture was heated for 30 min at 135 °C. The solvent was evaporated and the solid was washed with water (5 ml) and methanol (5 ml). The residue was purified via column chromatography (SiO2, toluene/PE 2:1). The pure fractions were evaporated under reduced pressure and washed with methanol (5 ml) yielding 13 (36.2 mg, 18.2 µmol, 70%) as a green solid.

^{1}H NMR (600.2 MHz, C_6D_6, 295K, mm06b/60012): δ[ppm]= 8.16 (s, 8H), 7.56 (t, J = 7.9 Hz, 8H), 7.47 (d, J= 7.9 Hz, 16H), 3.46 (sept, J= 6.8 Hz, 16H), 1.29 (broad-d, 96H).

^{13}C NMR (150.9 MHz, C_6D_6, 295K, mm06b/60010): δ[ppm]= 152.8 (s, C_q), 150.8 (s, C_q), 149.4 (s, C_q), 141.9 (s, C_q), 132.3 (s, C_q), 126.5 (s, CH_{Ar}), 124.9 (s, CH_{Ar}), 107.5 (s, CH_{Ar}), 27.6 (s, CH), 24.6 (bs, CH_3), 23.0 (bs, CH_3),

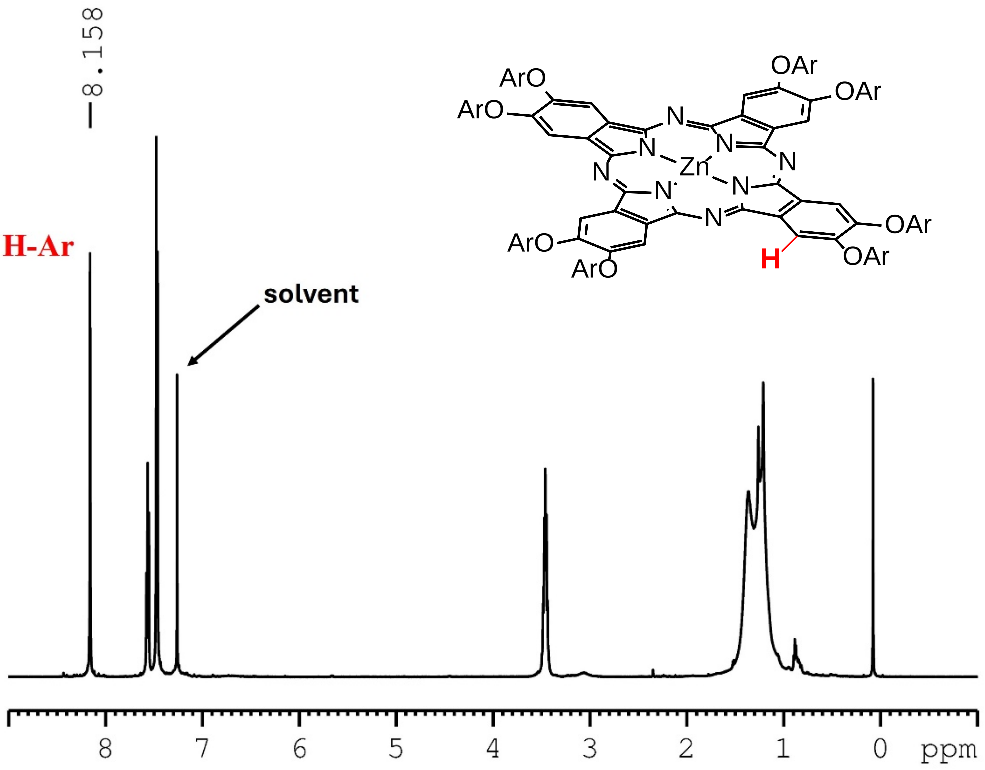

Figure S4: ^{1}H spectra of the corresponding Zn-complex to **1** in $CDCl_3$. (mm06b/60012)

Table S1. Magnetic-susceptibility anisotropies of selected diamagnetic compounds[1]. For the interpretation of the *RQC* data the magnetic anisotropy of the whole molecule has to be considered. In our case the eight phenoxy substituents are oriented orthogonal to the phthalocyanine so that the magnetic anisotropies cancel to a large extent.

Compound	$\Delta\chi_{dia}$ [10^{-32} m^3]
Porphyrin	-1.26
Benzene	-0.127
Toluene	-0.137

Table S2. Data extracted from NMR measurements (700 MHz) to plot Δ**χ**(*RQC*) and Δ**χ**(*PCS*).
*measured at 900MHz

NMR file name		T [K]	T^{-1} [10^{-3} K^{-1}]	*PCS* [ppm]	*RQC* [Hz]	Δ**χ**(*RQC*) [10^{-32} m^3]	Δ**χ**(*PCS*) [10^{-32} m^3]
lz04 (Me-Cy)*	95011	292.85	3.414717	14.38	119.82	-13.133	-11.225
lz04_icy	70301	303.0	3.30033	13.78	59.38	-12.404	-10.756
lz04_icy	70311	313.0	3.194888	13.24	57.41	-12.388	-10.335
lz04_icy	70321	323.0	3.095975	12.68	53.08	-11.819	-9.898
lz04_icy	70331	333.0	3.003003	12.18	48.72	-11.184	-9.508
lz04_icy	70341	343.0	2.915452	11.70	45.18	-10.684	-9.133
lz04_icy	70350	353.0	2.832861	11.21	42.85	-10.428	-8.750
lz04_icy	70360	363.0	2.754821	10.73	40.12	-10.0403	-8.376
lz04_icy	70370	373.0	2.680965	10.30	37.85	-9.733	-8.040
lz04_icy	70380	383.0	2.610966	9.94	37.19	-9.819	-7.759
lz04_icy	70390	393.0	2.544529	9.63	35.42	-9.596	-7.517
lz04_icy	70400	403.0	2.48139	9.30	32.94	-9.151	-7.260
lz04_icy	70410	413.0	2.421308	9.00	31.91	-9.086	-7.026
lz04_icy	70420	423.0	2.364066	8.72	29.80	-8.690	-6.807
lz04_icy	70430	433.0	2.309469	8.45	28.05	-8.372	-6.596
lz04_icy	70440	443.0	2.257336	8.23	26.47	-8.083	-6.425

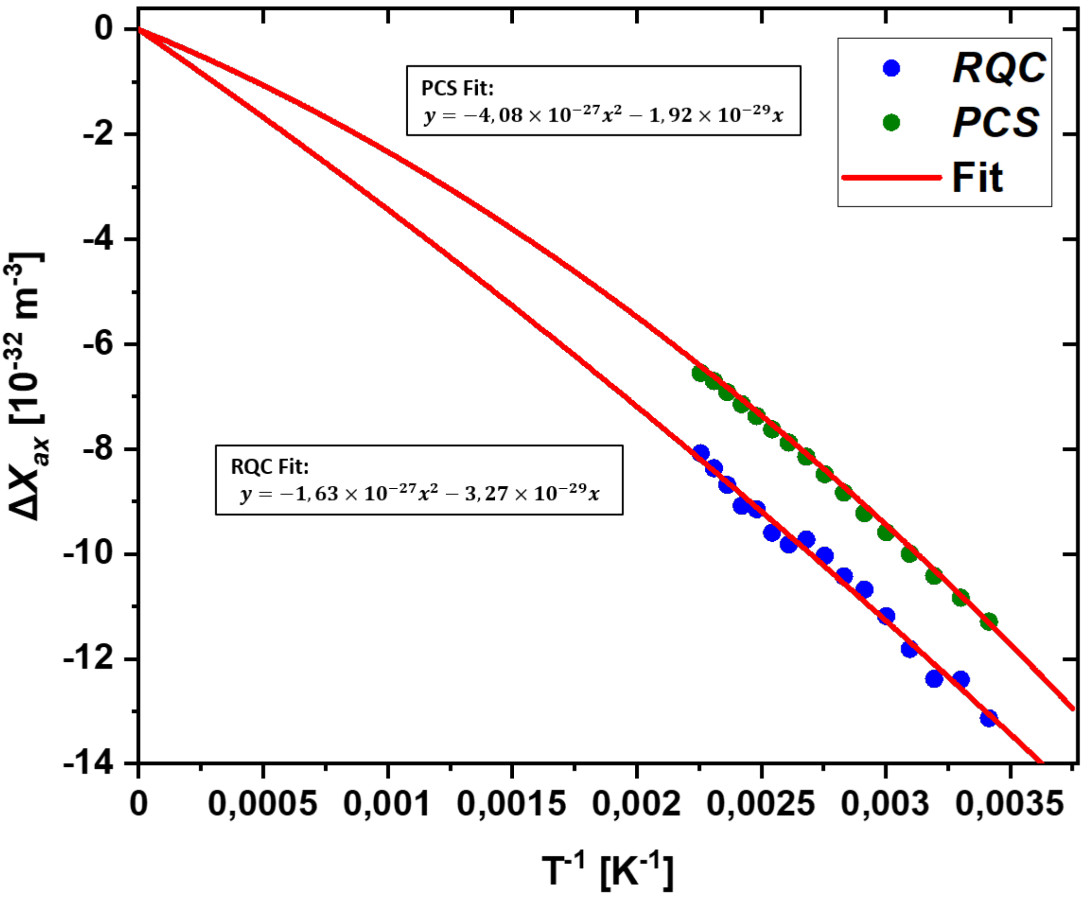

Figure S5 Fitting of $\Delta\chi$ from the experimental *pcs* and RQC data.

Evans method:

A diamagnetic correction for χ_{mol} was done by using the Pascal constants. For **1** we calculated $\chi_{mol,corr}$ as follows:

$$\chi_{mol,corr} = \chi_{mol} + 48\,C + 77\,C(Ring) + 8\,N(Ring) + 138\,H + 8\,O + Fe^{2+} = 5.85 \times 10^{-3}\,\frac{emu}{mol}$$

This results in μ_{eff} / μ_B = 3.7

Table S3. Pascal constants for different atoms in different molecular environments.[2]

Atom	Pascal constants [10^{-6} emu/mol]
C	-6.00
C(Ring)	-6.24
N	-5.57
N(Ring)	-4.61
H	-2.93
O	-4.6
Fe^{2+}	-13
Fe^{3+}	-10

2. Single Crystal X-Ray Diffraction

Crystal data and details of the structure determination are compiled in Table S4. Full shells of intensity data were collected at 120(1) K with an Agilent Technologies Supernova-E CCD diffractometer (Cu-K_α radiation, microfocus X-ray tube, multilayer mirror optics). Detector frames (typically ω-, occasionally φ-scans, scan width 1.0°) were integrated by profile fitting.[3] Data were corrected for air and detector absorption, Lorentz and polarization effects[4-5]and scaled essentially by application of appropriate spherical harmonic functions.[6-8]Absorption by the crystal was treated numerically (Gaussian grid).[8-9] An illumination correction was performed as part of the numerical absorption correction.[8]

Using OLEX2,[10] the structures were solved with SHELXT[11-12] (intrinsic phasing) and refined with SHELXL[11, 13-14] by full-matrix least squares methods based on F^2 against all unique reflections. All non-hydrogen atoms were given anisotropic displacement parameters. Hydrogen atoms were generally input at calculated positions and refined with a riding model.[15-16]

CCDC 2434366 contains the supplementary crystallographic data for this paper. These data can be obtained free of charge from the Cambridge Crystallographic Data Centre's and FIZ Karlsruhe’s joint Access Service via https://www.ccdc.cam.ac.uk.

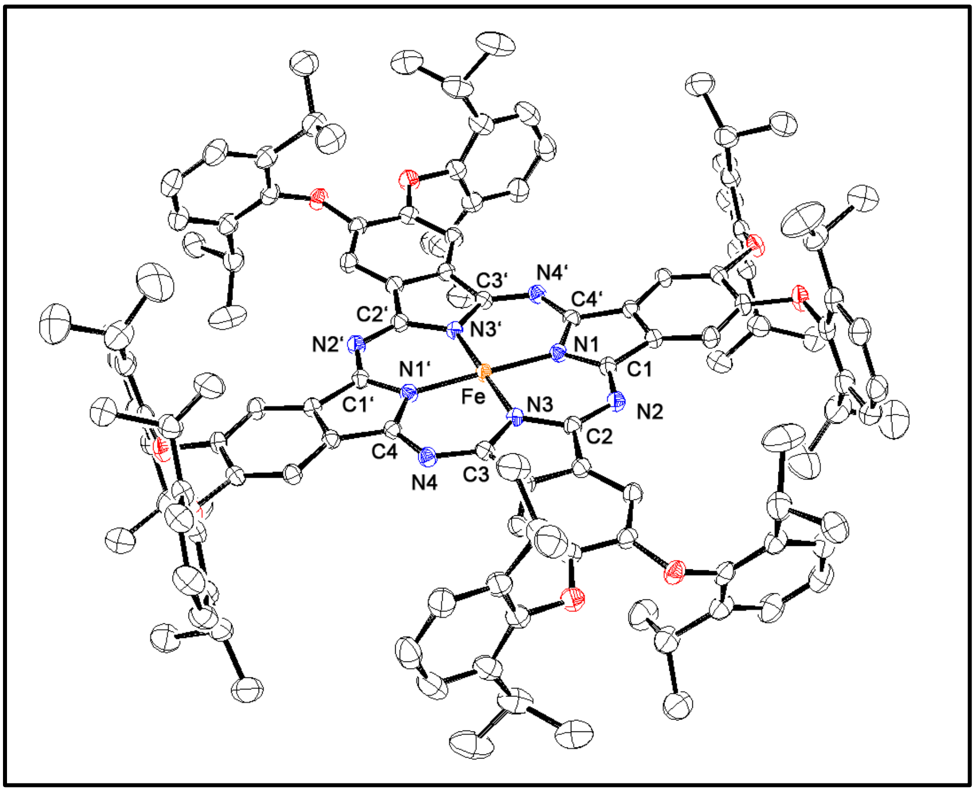

Figure S6. ORTEP plot of solid state molecular structure of **1**. Hydrogen atoms and solvent molecules (benzene) are omitted for clarity. Thermal ellipsoids are drawn at the 50% probability level.

Table S4. Bond Lengths for complex **1** (en_td3).

Atom	Atom	Length/Å		Atom	Atom	Length/Å
**Fe**	N1[1]	1.9350(12)		C23	C25	1.517(3)
**Fe**	N1	1.9350(12)		C26	C27	1.532(3)
**Fe**	N3[1]	1.9311(12)		C26	C28	1.526(3)
**Fe**	N3	1.9311(12)		C29	C30	1.397(2)
**O1**	C8	1.3794(17)		C29	C34	1.387(2)
**O1**	C29	1.4086(18)		C30	C31	1.394(2)
**O2**	C9	1.3687(17)		C30	C35	1.517(2)
**O2**	C17	1.4045(18)		C31	C32	1.384(3)
**O3**	C15	1.3756(18)		C32	C33	1.378(3)
**O3**	C41	1.4039(19)		C33	C34	1.396(3)
**O4**	C14	1.3729(18)		C34	C38	1.521(3)
**O4**	C53	1.395(2)		C35	C36	1.524(3)
**N1**	C1	1.3822(18)		C35	C37	1.533(3)
**N1**	C4[1]	1.3768(18)		C38	C39	1.510(3)
**N2**	C1	1.3262(19)		C38	C40	1.523(3)
**N2**	C2	1.3301(19)		C41	C42	1.398(2)
**N3**	C2	1.3790(18)		C41	C46	1.392(2)
**N3**	C3	1.3789(18)		C42	C43	1.393(3)
**N4**	C3	1.3208(19)		C42	C47	1.518(3)
**N4**	C4	1.3207(19)		C43	C44	1.382(3)
**C1**	C12[1]	1.461(2)		C44	C45	1.377(3)
**C2**	C5	1.455(2)		C45	C46	1.396(2)
**C3**	C6	1.442(2)		C46	C50	1.511(2)
**C4**	C11	1.444(2)		C47	C48	1.526(3)
**C5**	C6	1.390(2)		C47	C49	1.528(3)
**C5**	C10	1.402(2)		C50	C51	1.529(3)
**C6**	C7	1.396(2)		C50	C52	1.529(3)
**C7**	C8	1.378(2)		C53	C54	1.388(3)
**C8**	C9	1.421(2)		C53	C58	1.397(2)
**C9**	C10	1.383(2)		C54	C55	1.396(3)
**C11**	C12	1.393(2)		C54	C59	1.525(2)
**C11**	C16	1.397(2)		C55	C56	1.384(3)
**C12**	C13	1.400(2)		C56	C57	1.382(3)
**C13**	C14	1.391(2)		C57	C58	1.399(3)
**C14**	C15	1.414(2)		C58	C62	1.517(3)
**C15**	C16	1.379(2)		C59	C60	1.525(3)
**C17**	C18	1.395(2)		C59	C61	1.518(3)
**C17**	C22	1.397(2)		C62	C63	1.531(3)
**C18**	C19	1.398(2)		C62	C64	1.538(3)
**C18**	C23	1.516(2)		C65	C66	1.373(3)
**C19**	C20	1.377(3)		C65	C70	1.376(3)
**C20**	C21	1.386(3)		C66	C67	1.394(3)
**C21**	C22	1.394(2)		C67	C68	1.376(3)
**C22**	C26	1.522(2)		C68	C69	1.378(3)
**C23**	C24	1.521(3)		C69	C70	1.379(3)

Table S5. Bond Angles for complex **1** (en_td3).

Atom	Atom	Atom	Angle/˚		Atom	Atom	Atom	Angle/˚
**N1^1**	Fe	N1	180.0		C21	C22	C17	116.57(16)
**N3^1**	Fe	N1^1	89.26(5)		C21	C22	C26	122.76(15)
**N3^1**	Fe	N1	90.74(5)		C18	C23	C24	111.39(16)
**N3**	Fe	N1	89.26(5)		C18	C23	C25	111.33(14)
**N3**	Fe	N1^1	90.74(5)		C25	C23	C24	110.45(17)
**N3^1**	Fe	N3	180.0		C22	C26	C27	110.52(15)
**C8**	O1	C29	115.90(11)		C22	C26	C28	113.91(16)
**C9**	O2	C17	119.73(11)		C28	C26	C27	109.06(16)
**C15**	O3	C41	115.65(11)		C30	C29	O1	117.42(15)
**C14**	O4	C53	118.55(12)		C34	C29	O1	118.79(14)
**C1**	N1	Fe	127.18(10)		C34	C29	C30	123.79(15)
**C4^1**	N1	Fe	125.41(9)		C29	C30	C35	121.04(15)
**C4^1**	N1	C1	107.22(11)		C31	C30	C29	116.73(16)
**C1**	N2	C2	122.07(13)		C31	C30	C35	122.22(15)
**C2**	N3	Fe	127.13(10)		C32	C31	C30	120.96(16)
**C3**	N3	Fe	125.30(9)		C33	C32	C31	120.52(16)
**C3**	N3	C2	107.27(11)		C32	C33	C34	120.85(17)
**C3**	N4	C4	122.23(13)		C29	C34	C33	117.06(16)
**N1**	C1	C12^1	109.71(12)		C29	C34	C38	122.35(16)
**N2**	C1	N1	127.00(13)		C33	C34	C38	120.58(17)
**N2**	C1	C12^1	123.20(13)		C30	C35	C36	113.39(16)
**N2**	C2	N3	127.24(13)		C30	C35	C37	109.91(16)
**N2**	C2	C5	122.97(13)		C36	C35	C37	109.5(2)
**N3**	C2	C5	109.68(12)		C34	C38	C40	110.75(17)
**N3**	C3	C6	109.67(12)		C39	C38	C34	111.13(19)
**N4**	C3	N3	127.95(13)		C39	C38	C40	112.5(2)
**N4**	C3	C6	122.38(13)		C42	C41	O3	118.62(15)
**N1^1**	C4	C11	109.91(12)		C46	C41	O3	117.84(15)
**N4**	C4	N1^1	127.86(13)		C46	C41	C42	123.50(16)
**N4**	C4	C11	122.23(13)		C41	C42	C47	122.49(16)
**C6**	C5	C2	106.13(12)		C43	C42	C41	116.72(17)
**C6**	C5	C10	120.68(13)		C43	C42	C47	120.77(16)
**C10**	C5	C2	133.12(13)		C44	C43	C42	121.41(18)
**C5**	C6	C3	107.22(12)		C45	C44	C43	120.13(18)
**C5**	C6	C7	122.39(13)		C44	C45	C46	121.20(17)
**C7**	C6	C3	130.35(13)		C41	C46	C45	117.03(16)
**C8**	C7	C6	117.14(13)		C41	C46	C50	121.69(15)
**O1**	C8	C9	114.97(13)		C45	C46	C50	121.27(16)
**C7**	C8	O1	124.04(13)		C42	C47	C48	112.28(16)
**C7**	C8	C9	120.98(13)		C42	C47	C49	110.74(16)
**O2**	C9	C8	114.26(12)		C48	C47	C49	110.59(16)
**O2**	C9	C10	124.22(13)		C46	C50	C51	110.78(15)
**C10**	C9	C8	121.49(13)		C46	C50	C52	111.93(17)
**C9**	C10	C5	117.30(13)		C52	C50	C51	109.95(17)
**C12**	C11	C4	107.19(13)		O4	C53	C58	117.22(16)
**C12**	C11	C16	122.15(13)		C54	C53	O4	118.16(14)
**C16**	C11	C4	130.55(14)		C54	C53	C58	124.43(16)
**C11**	C12	C1^1	105.93(12)		C53	C54	C55	116.67(16)
**C11**	C12	C13	120.74(13)		C53	C54	C59	121.54(15)
**C13**	C12	C1^1	133.28(14)		C55	C54	C59	121.74(17)
**C14**	C13	C12	117.29(14)		C56	C55	C54	120.69(19)
**O4**	C14	C13	125.34(14)		C57	C56	C55	120.84(18)
**O4**	C14	C15	113.31(13)		C56	C57	C58	120.83(17)
**C13**	C14	C15	121.30(13)		C53	C58	C57	116.29(18)
**O3**	C15	C14	114.89(13)		C53	C58	C62	119.93(17)
**O3**	C15	C16	123.86(14)		C57	C58	C62	123.76(17)
**C16**	C15	C14	121.24(14)		C54	C59	C60	111.14(15)
**C15**	C16	C11	117.14(14)		C61	C59	C54	112.13(15)
**C18**	C17	O2	118.43(14)		C61	C59	C60	110.96(16)
**C18**	C17	C22	124.00(14)		C58	C62	C63	113.4(2)
**C22**	C17	O2	117.21(14)		C58	C62	C64	111.35(16)
**C17**	C18	C19	116.68(15)		C63	C62	C64	110.09(19)
**C17**	C18	C23	122.07(14)		C66	C65	C70	120.23(18)
**C19**	C18	C23	121.22(15)		C65	C66	C67	119.54(18)
**C20**	C19	C18	121.09(17)		C68	C67	C66	120.16(19)
**C19**	C20	C21	120.48(16)		C67	C68	C69	119.79(19)
**C20**	C21	C22	121.15(17)		C68	C69	C70	120.13(18)
**C17**	C22	C26	120.63(14)		C65	C70	C69	120.15(18)

Table S6. Crystal data and structure refinement for complex **1**.

**Identification code**	**en_td3**
**Empirical formula**	$C_{140}H_{156}N_8O_8Fe$
**Formula weight**	2134.57
**Temperature/K**	120(1)
**Crystal system**	monoclinic
**Space group**	$P2_1/c$
**a/Å**	21.3341(2)
**b/Å**	16.79220(10)
**c/Å**	18.2085(2)
**α/°**	90
**β/°**	112.1080(10)
**γ/°**	90
**Volume/Å^3**	6043.52(10)
**Z**	2
**ρ_{calc}g/cm^3**	1.173
**μ/mm^{-1}**	1.473
**F(000)**	2284.0
**Crystal size/mm^3**	0.22 × 0.09 × 0.07
**Radiation**	Cu Kα (λ = 1.54184)
**2Θ range for data collection/°**	6.908 to 142.364
**Index ranges**	-26 ≤ h ≤ 26, -20 ≤ k ≤ 20, -22 ≤ l ≤ 22
**Reflections collected**	138089
**Independent reflections**	11654 [R_{int} = 0.0681, R_{sigma} = 0.0246]
**Data/restraints/parameters**	11654/0/725
**Goodness-of-fit on F^2**	1.029
**Final R indexes [I>=2σ (I)]**	R_1 = 0.0418, wR_2 = 0.1129
**Final R indexes [all data]**	R_1 = 0.0460, wR_2 = 0.1166
**Largest diff. peak/hole / e Å^{-3}**	0.43/-0.26
**CCDC Number**	2434366

3. Mössbauer (additional data)

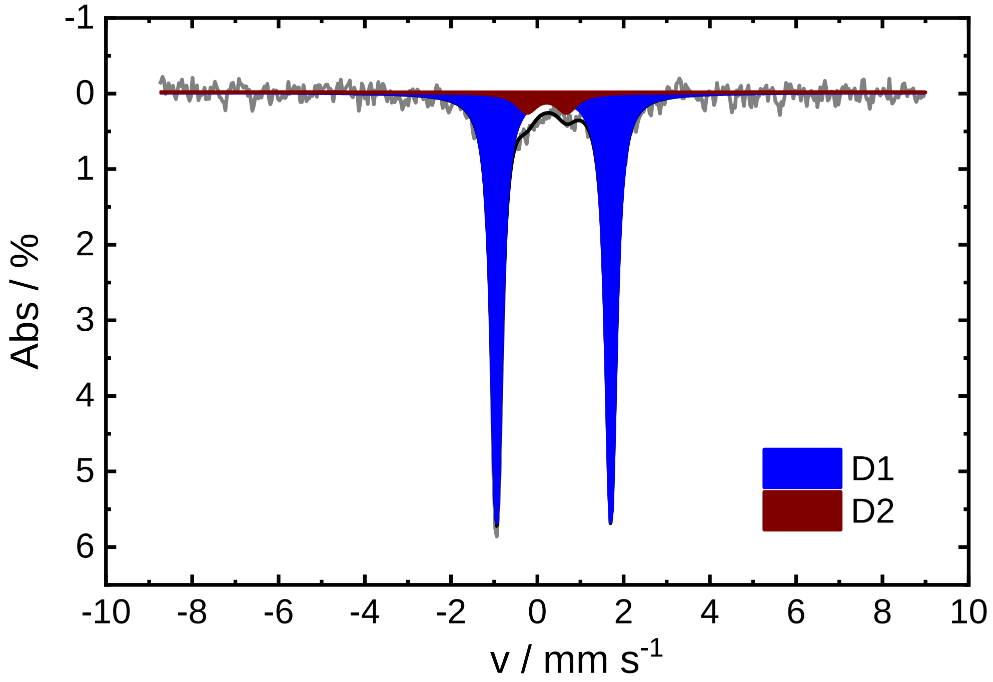

Figure S7 Mössbauer spectrum of the iron compound **1** obtained at 298K**.**

4. Calculations

Table S7 Excerpt from Gaussian output file of DFT calculation of **1** (UB3LYP/6-311Gd, S=1, file name: FePc_OAr_UB3LYP_6311Gd_D3_SP.out) showing the isotropic Fermi contact coupling. The yellow marked lines correspond to the eight H_{ar} atoms of **1**.

Isotropic Fermi Contact Couplings

	Atom	a.u.	MegaHertz	Gauss	10(-4) cm-1
1	Fe(57)	0.12694	9.20533	3.28469	3.07057
2	C(13)	-0.00166	-0.93418	-0.33334	-0.31161
3	C(13)	-0.00017	-0.09421	-0.03362	-0.03142
4	C(13)	0.00067	0.37733	0.13464	0.12586
5	C(13)	-0.00230	-1.29182	-0.46095	-0.43090
6	C(13)	-0.00101	-0.56521	-0.20168	-0.18853
7	C(13)	0.00049	0.27481	0.09806	0.09167
8	C(13)	0.00096	0.54232	0.19351	0.18090
9	H(1)	0.00011	0.25175	0.08983	0.08398
10	C(13)	-0.00097	-0.54505	-0.19449	-0.18181
11	H(1)	-0.00011	-0.23576	-0.08412	-0.07864
12	C(13)	-0.00168	-0.94466	-0.33708	-0.31511
13	C(13)	-0.00017	-0.09737	-0.03474	-0.03248
14	C(13)	0.00070	0.39340	0.14038	0.13122
15	C(13)	-0.00230	-1.29352	-0.46156	-0.43147
16	C(13)	-0.00101	-0.56574	-0.20187	-0.18871
17	C(13)	0.00047	0.26679	0.09520	0.08899
18	C(13)	0.00094	0.53042	0.18927	0.17693
19	H(1)	0.00011	0.24935	0.08897	0.08317
20	C(13)	-0.00095	-0.53550	-0.19108	-0.17862
21	H(1)	-0.00010	-0.22581	-0.08058	-0.07532
22	C(13)	-0.00173	-0.97046	-0.34629	-0.32371
23	C(13)	-0.00021	-0.12069	-0.04307	-0.04026
24	C(13)	0.00069	0.38737	0.13822	0.12921
25	C(13)	-0.00227	-1.27504	-0.45497	-0.42531
26	C(13)	-0.00099	-0.55767	-0.19899	-0.18602
27	C(13)	0.00048	0.26924	0.09607	0.08981
28	C(13)	0.00096	0.54241	0.19354	0.18093
29	H(1)	0.00011	0.24996	0.08919	0.08338
30	C(13)	-0.00094	-0.52713	-0.18809	-0.17583
31	H(1)	-0.00010	-0.22843	-0.08151	-0.07619
32	C(13)	-0.00023	-0.12926	-0.04612	-0.04312
33	C(13)	-0.00170	-0.95289	-0.34002	-0.31785
34	C(13)	-0.00224	-1.25998	-0.44959	-0.42028
35	C(13)	0.00068	0.38253	0.13649	0.12760
36	C(13)	0.00048	0.27200	0.09706	0.09073
37	C(13)	-0.00098	-0.54858	-0.19575	-0.18299
38	C(13)	-0.00094	-0.53113	-0.18952	-0.17716
39	H(1)	-0.00010	-0.23448	-0.08367	-0.07822
40	C(13)	0.00092	0.51624	0.18421	0.17220
41	H(1)	0.00011	0.24907	0.08888	0.08308
42	N(14)	0.00118	0.18986	0.06775	0.06333
43	N(14)	-0.00126	-0.20319	-0.07250	-0.06778
44	N(14)	0.00120	0.19371	0.06912	0.06461
45	N(14)	-0.00121	-0.19505	-0.06960	-0.06506
46	N(14)	-0.03070	-4.95938	-1.76963	-1.65427
47	N(14)	-0.03092	-4.99488	-1.78230	-1.66611
48	N(14)	-0.03079	-4.97407	-1.77487	-1.65917
49	N(14)	-0.03102	-5.01215	-1.78846	-1.67187
50	O(17)	-0.00034	0.10165	0.03627	0.03391
51	O(17)	0.00049	-0.14793	-0.05278	-0.04934
52	O(17)	0.00051	-0.15366	-0.05483	-0.05126
53	O(17)	-0.00030	0.09058	0.03232	0.03021
54	O(17)	0.00052	-0.15909	-0.05677	-0.05307
55	O(17)	0.00064	-0.19410	-0.06926	-0.06474
56	O(17)	-0.00026	0.07971	0.02844	0.02659
57	O(17)	-0.00027	0.08105	0.02892	0.02704

Table S8: Cartesian coordinates of FePc.

Fe	4,89448494449847	2,37874156109039	8,22599228168933
C	2,62942800010719	0,45711942230489	8,01918574317939
C	1,44654115291778	0,01428020951149	8,73752064872845
C	0,46866748263421	-0,94705578752050	8,44332699920180
H	0,51865774295386	-1,52949354700304	7,51902268262016
C	-0,56197161757641	-1,12479647615403	9,37373051972842
H	-1,34491341311965	-1,86565104983082	9,18026022425616
C	-0,61311210247825	-0,36277054005770	10,56461530158050
H	-1,43521498612156	-0,52996052900154	11,26870776339800
C	0,36391215125470	0,59696251268932	10,85722200716850
H	0,33160852577626	1,19072119008760	11,77503608699050
C	1,39506966017665	0,77411821884894	9,92514464354925
C	2,54855214741027	1,66071646575578	9,89980834340074
C	3,85782777015186	3,32546156339823	10,84271507567370
C	4,14854388911715	4,27196022540616	11,90888452988650
C	3,49251123459614	4,56555746424827	13,11180663296180
H	2,57805412881293	4,03601259230301	13,39350463746210
C	4,05329659160797	5,55584532687360	13,92788280533530
H	3,56949251030491	5,81345404722191	14,87610111970400
C	5,23673638407124	6,23443068812145	13,55296793720010
H	5,64465786953003	7,00273172770864	14,21824731230610
C	5,89117095100961	5,94030165765000	12,35094887058740
H	6,80667157801283	6,45765980105184	12,05056111216870
C	5,32862761611959	4,94845233701788	11,53453931465290
C	5,72675157410346	4,39716004905077	10,25043601108720
N	3,27710031626028	1,45086059318164	8,74036818606860
N	2,80907991008630	2,52002182839943	10,87089830326780
N	4,82383683705533	3,41993999452821	9,85419081485842
N	6,80401956284999	4,80767034755559	9,60164298067063
C	7,15955656259317	4,30034915577489	8,43283066602889
C	8,34253893013083	4,74307774032141	7,71456312064786
C	9,32045479411585	5,70435546843993	8,00880800957285
H	9,27047457184200	6,28677063096003	8,93312628599134
C	10,35112495693910	5,88205263404380	7,07842653180173
H	11,13410332823280	6,62286216421313	7,27192422508962
C	10,40224868985880	5,12005376020416	5,88752896247372
H	11,22437483407730	5,28721859535291	5,18345709206373
C	9,42517676123986	4,16038073338496	5,59487100268488
H	9,45746622588190	3,56664649563230	4,67704148478029
C	8,39399288753746	3,98326677258811	6,52692821928083
C	7,24041973998142	3,09677091965785	6,55218390535428
C	5,93113975904513	1,43202496935530	5,60928470269223
C	5,64040555357341	0,48554364012014	4,54310516725846
C	6,29649580176602	0,19186247171978	3,34023622264379
H	7,21096556196172	0,72138395673809	3,05853515255448
C	5,73575258250422	-0,79848357877332	2,52420238120805
H	6,21960940787964	-1,05615831364122	1,57602880842599
C	4,55229964045572	-1,47705213908269	2,89910701770039
H	4,14441897119708	-2,24540476958448	2,23386223908375
C	3,89781053292048	-1,18284705108574	4,10107727042714
H	2,98230002789926	-1,70018316966770	4,40147249293604
C	4,46031041887827	-0,19093296283841	4,91744172908468
C	4,06220482655828	0,36033959177124	6,20155868218611
N	6,51182863134738	3,30670632926511	7,71157438646763
N	6,97986381852635	2,23749044745523	5,58108603249668
N	4,96514777301873	1,33752423158552	6,59782730038029
N	2,98492799791428	-0,05017161834785	6,85034201930073

Table S9: Cartesian coordinates FePc^{OAr}.

Fe	0.000179205	-0.000318238	-0.000100479
O	6.616701481	-4.249761897	0.430418138
O	4.908561902	-6.142894470	0.192112358
O	6.170115019	4.860496257	0.118659488
O	4.280108415	6.600750428	0.160749374
N	1.432882989	-1.300197864	0.100923592
N	1.283591073	1.442088131	0.215521246
N	3.353167953	0.174308234	0.266736578
N	-0.184626122	3.368429269	0.135640553
C	2.775422032	-1.019015581	0.212837739
C	3.539588232	-2.256585030	0.212295905
C	4.902449794	-2.507029107	0.353020504
H	5.527022349	-1.800584028	0.468481942
C	5.306372533	-3.828071482	0.317370261
C	4.372683867	-4.873364490	0.151376755
C	3.020483051	-4.617507996	0.002914902
H	2.394071899	-5.320532523	-0.123078619
C	2.617432385	-3.283510646	0.046255179
C	1.305032009	-2.661908912	-0.014931972
C	2.648920856	1.300008923	0.259927684
C	3.279075014	2.607479953	0.282428362
C	4.623718553	2.983091891	0.269531960
H	5.319381267	2.336847847	0.299841539
C	4.897692652	4.333373223	0.210857941
C	3.853600860	5.292549277	0.203298002
C	2.524308621	4.915558347	0.224693368
H	1.826711827	5.560409727	0.218807858
C	2.249123089	3.543758960	0.255386136
C	1.007918071	2.787880213	0.205509832
C	7.599689367	-3.270388212	0.602708394
C	8.119395194	-2.642024155	-0.523699309
C	9.155977613	-1.729235045	-0.295287779
H	9.528245289	-1.257469118	-1.031051946
C	9.637674779	-1.503746785	0.939319771
H	10.357734242	-0.896420356	1.062470820
C	9.101535173	-2.137822832	2.006800610
H	9.458868306	-1.964661678	2.869830581
C	8.046882678	-3.033233252	1.885507991
C	7.617558806	-2.940967095	-1.905879254
H	6.807850099	-3.518153946	-1.799867233
C	7.163048313	-1.672480365	-2.633130798
H	6.493878843	-1.206361706	-2.089674511
H	6.770550720	-1.914553741	-3.497844033
H	7.933812867	-1.084659742	-2.777320576
C	8.624339361	-3.743079424	-2.713139840
H	9.455816172	-3.231574466	-2.799237372
H	8.258570524	-3.924325532	-3.604068187
H	8.807986129	-4.590662449	-2.256785728
H	7.164866653	-2.287705352	4.405025479
C	7.422609446	-3.736736714	3.084139113
H	6.443626791	-3.783145451	2.885545587
C	7.575996723	-2.608109263	4.247360883
H	8.514106554	-2.550433173	4.524894235
H	7.022171678	-2.855062636	5.017205145
C	7.796623497	-4.953049968	3.244604055
H	7.674739442	-5.445600325	2.406181820
H	7.258516769	-5.369970815	3.949605973
H	8.742379139	-4.972910583	3.500620710
C	4.233881996	-7.158356782	-0.494698523
C	3.311014569	-7.916235835	0.220940597
C	2.722443942	-8.986458380	-0.452001757
H	2.074308295	-9.524114399	-0.012299339
C	3.078472898	-9.265373171	-1.751434392
H	2.676332461	-10.000431785	-2.199173557
C	4.005863853	-8.497652343	-2.410610496
H	4.240590769	-8.719597695	-3.303997685
C	4.607160366	-7.408280460	-1.805361196
C	3.008286700	-7.605018021	1.660244643
H	3.153489707	-6.624019595	1.788837037
C	1.556955152	-7.904636400	2.047526385
H	0.954131414	-7.522742409	1.375843072
H	1.363925842	-7.508256801	2.922755048
H	1.424252013	-8.874605118	2.091776188
C	3.982135126	-8.331306160	2.581071480
H	3.878022941	-9.299224022	2.468536103
H	3.794433349	-8.088587439	3.511808426
H	4.900016082	-8.072999195	2.354848362
C	5.615658670	-6.491838626	-2.499651443
H	6.093994627	-6.009421845	-1.765859139
C	4.886823487	-5.415628644	-3.295801569
H	4.302392439	-5.839155941	-3.958710595
H	5.541684798	-4.847742779	-3.753045248
H	4.347809276	-4.868032393	-2.687533778
C	6.656293212	-7.178892856	-3.268018679
H	7.094384616	-7.846778842	-2.700198265
H	7.319467387	-6.526403398	-3.576012602
H	6.251290146	-7.623206004	-4.041955008
C	7.226234237	3.937262102	0.034872924
C	7.898428556	3.635548042	1.220325265
C	8.978578782	2.761346248	1.125182406
H	9.460057960	2.523537733	1.908831446
C	9.360158986	2.233447589	-0.100227100
H	10.102309390	1.642223475	-0.146851876
C	8.675008585	2.557354638	-1.247630163
H	8.951935789	2.187008167	-2.077481912
C	7.578042555	3.422402860	-1.212965070
C	7.471264946	4.294443740	2.524243050
H	6.471534610	4.305474960	2.544684486
C	7.951108842	3.553545557	3.769988127
H	8.928576766	3.600839562	3.821985671
H	7.671635768	2.615551878	3.720128305
H	7.561079401	3.968640557	4.567477668
C	7.951650101	5.747561646	2.555289498
H	7.650658381	6.173465811	3.384991800
H	7.581288364	6.230526829	1.787187281
H	8.930527511	5.768968310	2.513558462
C	6.810959039	3.761836972	-2.475963516
H	6.101196893	4.422686447	-2.232041825
C	6.113899395	2.518029588	-3.048386770
H	6.788682150	1.860760649	-3.318690201
H	5.576781214	2.773912698	-3.827106761
H	5.531650189	2.126059751	-2.364471901
C	7.707844196	4.411340456	-3.532872713
H	8.075926836	5.248029274	-3.179484750
H	7.180757807	4.600304408	-4.337155033
H	8.441014390	3.801518386	-3.758608566
C	3.382768056	7.556861212	-0.343655825
C	2.830450108	8.452428149	0.569471433
C	1.996990613	9.443622730	0.042633920
H	1.599244001	10.079614807	0.625582678
C	1.743590184	9.511213255	-1.314396033
H	1.172320680	10.190317441	-1.653469788
C	2.312180757	8.602419513	-2.179574029
H	2.121877233	8.659849253	-3.108541824
C	3.161520145	7.600614273	-1.715242999
C	3.158715328	8.341137136	2.048620251
H	3.057874221	7.379863042	2.305074169
C	4.616773852	8.738069861	2.323189038
H	5.212529492	8.207217668	1.754287580
H	4.829207888	8.570181602	3.265037504
H	4.738927128	9.690109995	2.125449441
C	2.220912583	9.154384883	2.946252569
H	2.348248465	10.110139707	2.770875726
H	2.422909444	8.965018137	3.886323444
H	1.291627241	8.908298816	2.755817178
C	3.856972649	6.631093887	-2.657782764
H	4.237843321	5.901568161	-2.089691297
C	5.030755871	7.307123656	-3.344354126
H	4.698758486	8.002976901	-3.949303349
H	5.536249455	6.642403037	-3.857211764
H	5.614712685	7.712259050	-2.669631524
C	2.926328197	5.976509565	-3.631548476
H	2.179492002	5.568306323	-3.145774006
H	3.408996605	5.284476038	-4.130041835
H	2.581501219	6.648440182	-4.256054790
O	-6.616342541	4.249126135	-0.430618638
O	-4.908203492	6.142257994	-0.192313315
O	-6.169757143	-4.861132033	-0.118860919
O	-4.279750667	-6.601385483	-0.160949595
N	-1.432525771	1.299562095	-0.101124270
N	-1.283233196	-1.442723906	-0.215722677
N	-3.352810077	-0.174944009	-0.266938009
N	0.184984529	-3.369064331	-0.135841527
C	-2.775064815	1.018379812	-0.213038417
C	-3.539230356	2.255949255	-0.212497336
C	-4.902091918	2.506393331	-0.353221935
H	-5.526665131	1.799948260	-0.468682621
C	-5.306015315	3.827435713	-0.317570939
C	-4.372326649	4.872728721	-0.151577433
C	-3.020124645	4.616872934	-0.003115875

H	-2.393714682	5.319896754	0.122877940
C	-2.617074633	3.282874177	-0.046455384
C	-1.304674257	2.661272443	0.014731767
C	-2.648562446	-1.300645399	-0.260128642
C	-3.278716073	-2.608115715	-0.282628862
C	-4.623361336	-2.983727659	-0.269732638
H	-5.319023391	-2.337483623	-0.300042970
C	-4.897335434	-4.334008992	-0.211058620
C	-3.853242984	-5.293185053	-0.203499433
C	-2.523950873	-4.916193402	-0.224893589
H	-1.826354610	-5.561045496	-0.219008536
C	-2.248764683	-3.544394022	-0.255587109
C	-1.007559660	-2.788516689	-0.205710789
C	-7.599331491	3.269752436	-0.602909825
C	-8.119036253	2.641388393	0.523498810
C	-9.155620395	1.728599276	0.295087101
H	-9.527888071	1.256833349	1.030851268
C	-9.637316369	1.503110309	-0.939520728
H	-10.357375832	0.895783880	-1.062671777
C	-9.101177421	2.137186363	-2.007000815
H	-9.458509896	1.964025202	-2.870031538
C	-8.046524268	3.032596776	-1.885708948
C	-7.617200396	2.940330619	1.905678297
H	-6.807492881	3.517518177	1.799666555
C	-7.162690565	1.671845310	2.632930577
H	-6.493521625	1.205725937	2.089473832
H	-6.770193502	1.913917972	3.497643354
H	-7.933454461	1.084024680	2.777119603
C	-8.623980955	3.742444363	2.712938867
H	-9.455457231	3.230938704	2.799036872
H	-8.258212114	3.923689056	3.603867229
H	-8.807628253	4.590026673	2.256584297
C	-7.422251569	3.736100939	-3.084340544
H	-6.794582373	4.406499328	-2.688607171
H	-7.164508247	2.287070290	-4.405226453
C	-7.575638312	2.607472787	-4.247561840
H	-8.513748677	2.549797398	-4.525095666
H	-7.021813802	2.854426860	-5.017406575
C	-7.796265087	4.952413492	-3.244805012
H	-7.674382224	5.444964556	-2.406382499
H	-7.258158363	5.369335753	-3.949806947
H	-8.742020857	4.972274828	-3.500820518
C	-4.233523586	7.157720306	0.494497566
C	-3.310656162	7.915600773	-0.221141570
C	-2.722085536	8.985823318	0.451800783
H	-2.073951078	9.523478630	0.012098661
C	-3.078114492	9.264738109	1.751233419
H	-2.675974585	9.999796009	2.198972126
C	-4.005506635	8.497016575	2.410409818
H	-4.240232893	8.718961919	3.303796254
C	-4.606803148	7.407644691	1.805160517
C	-3.007927759	7.604382259	-1.660445143
H	-3.153131297	6.623383119	-1.789037994
C	-1.556597934	7.904000631	-2.047727064
H	-0.953773538	7.522106634	-1.376044503
H	-1.363568625	7.507621033	-2.922955726
H	-1.423893603	8.873968642	-2.091977145
C	-3.981776716	8.330669685	-2.581272437
H	-3.877665064	9.298588247	-2.468737534
H	-3.794076131	8.087951670	-3.512009104
H	-4.899658206	8.072363420	-2.355049793
C	-5.615300794	6.491202851	2.499450012
H	-6.093636217	6.008785369	1.765658182
C	-4.886465080	5.414993582	3.295600595
H	-4.302035222	5.838520173	3.958509917
H	-5.541327581	4.847107010	3.752844570
H	-4.347450994	4.867396637	2.687334031
C	-6.655934806	7.178257794	3.267817706
H	-7.094027398	7.846143073	2.699997587
H	-7.319108977	6.525766922	3.575811645
H	-6.250932399	7.622570949	4.041754787
C	-7.225876360	-3.937897877	-0.035074355
C	-7.898070679	-3.636183818	-1.220526696
C	-8.978221564	-2.761982017	-1.125383084
H	-9.459700742	-2.524173502	-1.909032124
C	-9.359801234	-2.234084058	0.100026896
H	-10.101952173	-1.642859244	0.146651197
C	-8.674650178	-2.557989699	1.247429190
H	-8.951577912	-2.187643943	2.077280481
C	-7.577684679	-3.423038636	1.212763639
C	-7.470907070	-4.295079515	-2.524444481
H	-6.471176199	-4.306111436	-2.544885443
C	-7.950750966	-3.554181333	-3.770189558
H	-8.928218890	-3.601475338	-3.822187102
H	-7.671277891	-2.616187654	-3.720329736
H	-7.560721525	-3.969276333	-4.567679099
C	-7.951292353	-5.748196701	-2.555489719
H	-7.650300504	-6.174101586	-3.385193231
H	-7.580931146	-6.231162598	-1.787387959
H	-8.930169635	-5.769604086	-2.513759893
C	-6.810601163	-3.762472747	2.475762086
H	-6.100839016	-4.423322223	2.231840395
C	-6.113541518	-2.518665363	3.048185339
H	-6.788324398	-1.861397118	3.318489996
H	-5.576423996	-2.774548467	3.826906082
H	-5.531291783	-2.126694813	2.364270928
C	-7.707486445	-4.411976926	3.532672508
H	-8.075568960	-5.248665050	3.179283319
H	-7.180399400	-4.600939470	4.336954060
H	-8.440656642	-3.802153441	3.758408345
C	-3.382410179	-7.557496988	0.343454394
C	-2.830092231	-8.453063924	-0.569672864
C	-1.996631673	-9.444258492	-0.042834420
H	-1.598885595	-10.080249868	-0.625783652
C	-1.743232966	-9.511849024	1.314195355
H	-1.171962270	-10.190953917	1.653268830
C	-2.311823009	-8.603054568	2.179373808
H	-2.121519356	-8.660485028	3.108340393
C	-3.161161735	-7.601250749	1.715042042
C	-3.158357576	-8.341773605	-2.048820456
H	-3.057515811	-7.380499518	-2.305275126
C	-4.616416635	-8.738705630	-2.323389717
H	-5.212171616	-8.207853444	-1.754489011
H	-4.828849478	-8.570818078	-3.265238461
H	-4.738568188	-9.690745757	-2.125649940
C	-2.220555366	-9.155020652	-2.946453247
H	-2.347890055	-10.110776183	-2.771076683
H	-2.422551568	-8.965653913	-3.886524875
H	-1.291268831	-8.908935292	-2.756018135
C	-3.856614239	-6.631730363	2.657581807
H	-4.237485445	-5.902203937	2.089489866
C	-5.030398124	-7.307758711	3.344153905
H	-4.698400079	-8.003611963	3.949102376
H	-5.535892237	-6.643038806	3.857011086
H	-5.614354279	-7.712894112	2.669430551
C	-2.925970979	-5.977145334	3.631347798
H	-2.179134126	-5.568942099	3.145572575
H	-3.408638853	-5.285112507	4.129841630
H	-2.581142813	-6.649075244	4.255853817

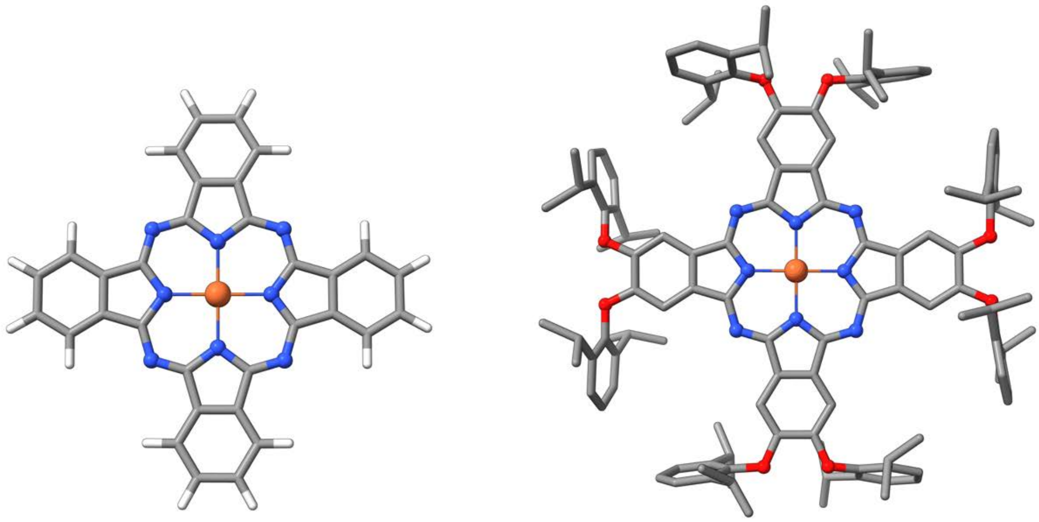

Figure S 8.Calculated bond distances and angles for FePc (left) and $FePc^{OAr}$ (right). The H atoms in $FePc^{OAr}$ have been omitted in $FePc^{OAr}$ for clarity. [$FePc^{OAr}$]: Fe-N_1 = 1.937(15)Å, Fe-N_2 = 1.942(72)Å, Fe-N_3 = 1.937(15)Å, Fe-N_4 = 1.942(72)Å ; [FePc]: Fe-N_1 = 1.934(29)Å, Fe-N_2 = 1.933(94)Å, Fe-N_3 = 1.934(31)Å, Fe-N_4 = 1.933(92)Å). N-Fe-N angles ([$FePc^{OAr}$] = [FePc]: $N_{1/3}$-Fe-$N_{2/4}$ = 90.2(20), $N_{2/4}$-Fe-$N_{3/1}$ = 89,7(80).

CASSCF active spaces

To assess the robustness of the computed magnetic properties, several CASSCF/NEVPT2 calculations were carried out using different active spaces. Most of the attempted active spaces failed to converge, particularly those including different Gouterman-like orbitals. An active space of 18 electrons and 14 orbitals (without the $d_{x^2-y^2}$ orbital due to its high energy in square-planar conformations) yielded magnetic parameters (g-tensor and zero-field splitting D) that were less accurate compared to larger space which includes the $d_{x^2-y^2}$ orbital (Figure S9, S10 and Table S13) A total of 100 states were calculated from this space for the three spin multiplicites (5 quintets, 45 triplets and 50 singlets) including all the possible CSFs arising from a d^6 configuration as stated by Sing, et al [17]. This result suggests that the magnetic properties are not significantly affected by the exclusion of this orbital nor the inclusion of higher energy excitations.

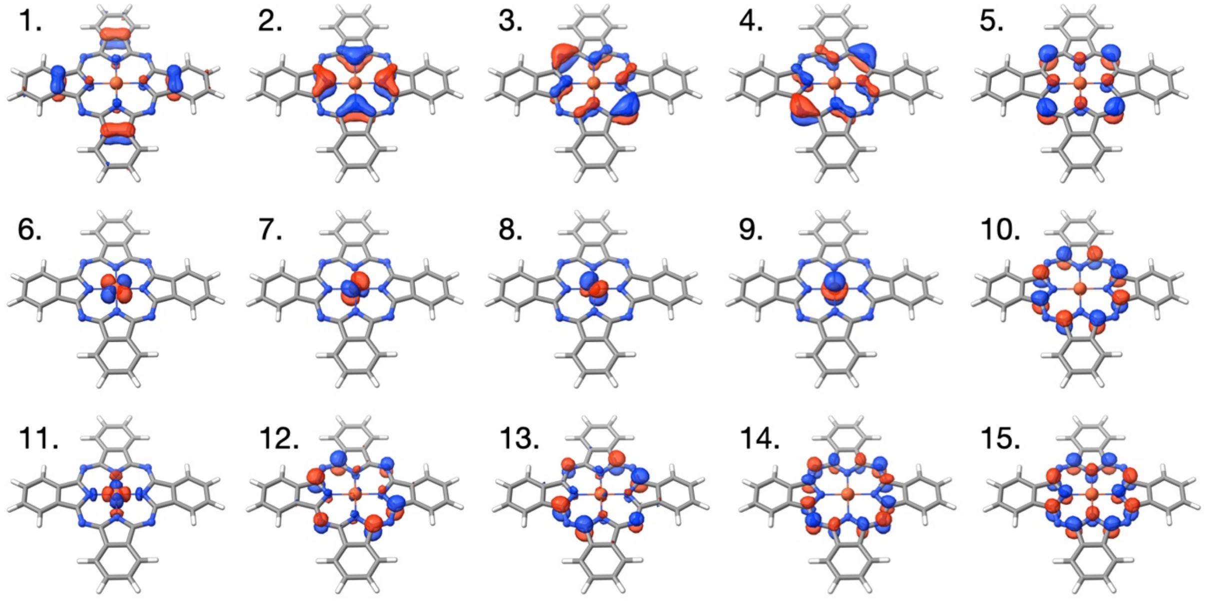

Figure S9. State average active space (18e - 15o). SA-CASCCF/NEVPT2-DKH-SOC | 60 states, Fe – def2-QZVPP | N and C – def2-TZVP | H – def2-SVP

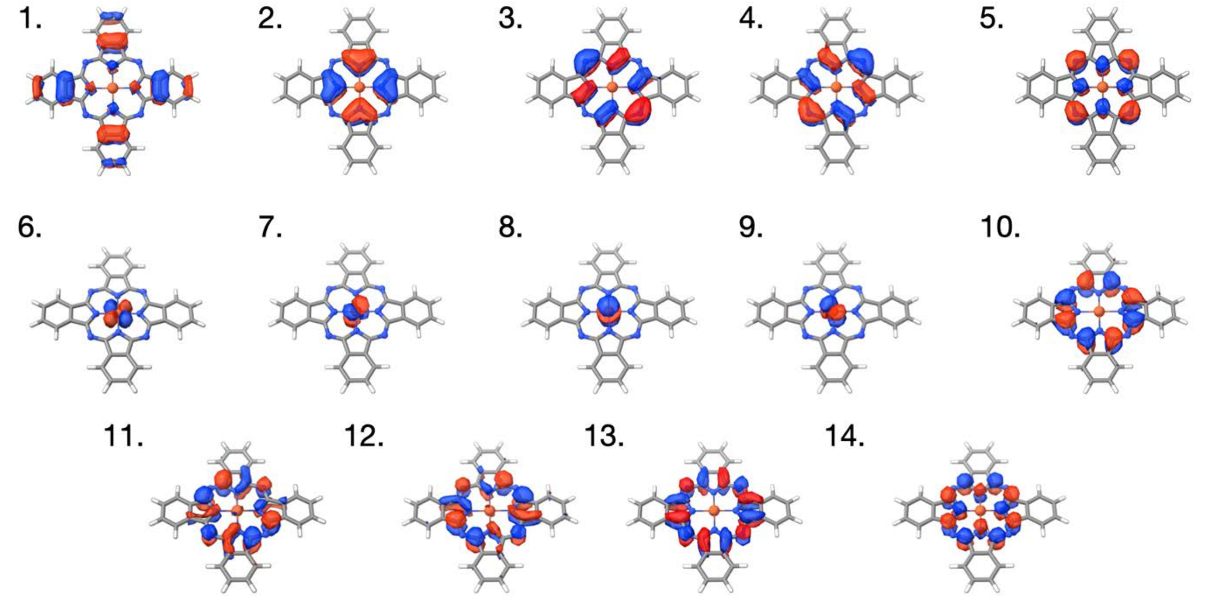

Figure S10. State average active space (18e - 14o). SA-CASCCF/NEVPT2-DKH-SOC | 100 states, Fe – def2-QZVPP | N and C – def2-TZVP | H – def2-SVP

Table S10. CASSCF/NEVPT2 states and configuration weights for the state average active space (18e - 15o).

		ΔE$_{state}$			
ROOT	MULT	CASSCF(18,15)	NEVPT2	Weight	Configuration
0	5	0.0	4102.3	0.83710	222221112210000
1	5	1727.3	5380.4	0.83699	222221211210000
2	5	1753.3	5388.4	0.83693	222221121210000
3	5	3652.9	6801.1	0.83726	222222111210000
4	5	10680.0	14416.9	0.87541	222221112111000
5	5	10787.8	14478.5	0.87367	222221112110100
6	5	10933.4	13355.0	0.43289	222221121111000
				0.40648	222221211110100
7	5	13528.0	17738.0	0.73904	222221211111000
8	5	13663.4	17660.3	0.46647	222221211110100
				0.44142	222221121111000
9	5	13775.0	17904.3	0.73959	222221121110100
10	5	14347.5	16922.7	0.86434	222222111111000
11	5	14456.6	16902.1	0.84985	222222111110100
12	5	14893.3	11460.5	0.86294	222222112101000
13	5	15010.3	11455.4	0.87499	222222112100100
14	5	15353.1	11820.6	0.55768	222222121101000
15	5	15600.3	12362.5	0.60071	222222211101000
16	5	15651.2	12247.7	0.54927	222222211100100
17	5	15805.8	12575.6	0.60192	222222121100100
18	5	20209.8	16840.9	0.81449	222221221101000
19	5	20331.6	16935.2	0.81382	222221221100100
0	3	3757.3	0.0	0.81330	222222112200000
1	3	4243.5	591.6	0.71554	222222121200000
2	3	4253.1	600.1	0.71091	222222211200000
3	3	8832.2	5090.9	0.75764	222221221200000
4	3	10000.5	12000.6	0.41427	222221121111000
				0.39739	222221211110100
5	3	10482.9	14026.5	0.85724	222221112111000
6	3	10586.0	14078.7	0.85540	222221112110100
7	3	13402.4	8410.1	0.38171	222222121101000
				0.34378	222222211100100
8	3	13827.7	18131.0	0.82416	222221211111000
9	3	14016.7	18188.7	0.43841	222221211110100
				0.42657	222221121111000
10	3	14020.5	18257.5	0.78522	222221121110100
11	3	14123.3	16098.2	0.79561	222222111111000
12	3	14159.9	11368.3	0.63963	222222112101000
				0.19462	222222111110100
13	3	14269.6	15148.7	0.65878	222222111110100
				0.19030	222222112101000
14	3	14302.3	10372.5	0.77164	222222112100100
15	3	15083.7	11267.0	0.53156	222221212200000
				0.14855	222221121210000
16	3	15119.3	11300.2	0.53339	222221122200000
				0.15000	222221211210000
17	3	15692.8	12213.4	0.37167	222222211100100
				0.34004	222222121101000
18	3	15953.0	12823.5	0.67815	222222211101000
				0.10419	222221212101000
19	3	16105.9	12916.8	0.68206	222222121100100
				0.10050	222221122100100
0	1	12701.3	7375.2	0.37133	222222121101000

				0.34321	222222211100100
1	1	14031.1	9548.0	0.82101	222222112101000
2	1	14133.2	9595.2	0.81973	222222112100100
3	1	14698.1	9850.6	0.71533	222222220200000
4	1	15201.4	10413.5	0.76753	222222211200000
5	1	15206.0	10410.4	0.76904	222222121200000
6	1	15412.5	10498.2	0.76247	222222112200000
7	1	15699.9	12137.3	0.35080	222222211100100
				0.32903	222222121101000
8	1	16130.0	13034.3	0.72663	222222211101000
9	1	16262.2	13094.2	0.73143	222222121100100
10	1	16726.1	10842.0	0.37308	222222202200000
				0.37071	222222022200000
11	1	20277.6	16960.5	0.80894	222221221101000
12	1	20401.1	17058.2	0.80817	222221221100100
13	1	23472.8	17845.0	0.25418	222222022200000
				0.25392	222222202200000
14	1	24093.2	18442.7	0.65708	222221221200000
15	1	24437.1	18830.1	0.24569	222221122101000
				0.22856	222221212100100
16	1	25658.3	20653.1	0.56597	222221212200000
17	1	25692.0	20678.0	0.56694	222221122200000
18	1	26213.1	23351.2	0.30266	222221212101000
19	1	26675.4	23293.6	0.30726	222221212100100
				0.29279	222221122101000

Table S11. QDPT-SOC-CASSCF/NEVPT2 states. Only the first 50 states displayed

	ΔE_{state}	
Eigenvalues	CASSCF(18,15)	NEVPT2
0	0.00	0.00
1	26.34	90.18
2	27.47	91.98
3	108.48	658.67
4	108.77	717.30
5	1673.89	1047.23
6	1673.93	1053.06
7	1750.96	1053.91
8	1755.95	1278.83
9	1839.99	4378.85
10	1863.14	4380.61
11	1934.25	4382.05
12	2023.20	4389.09
13	2122.93	4390.07
14	2123.13	5160.07
15	3564.64	5160.44
16	3661.84	5289.74
17	3664.70	5646.63
18	3712.57	5705.60
19	3754.28	5706.42
20	3857.18	5827.20
21	3865.99	5840.33
22	3870.88	5845.83
23	4235.03	5848.65
24	4357.39	5938.85
25	4687.21	5969.77
26	4693.33	5970.55
27	4711.32	7159.47
28	4925.21	7159.89
29	8993.94	7176.90
30	8994.17	7177.62
31	9010.93	7182.06
32	9964.75	7637.45
33	10037.40	8626.86
34	10048.58	8631.36
35	10557.26	8669.68
36	10574.29	9837.37
37	10594.24	9880.10
38	10630.10	10218.48
39	10632.49	10594.53
40	10676.13	10616.20
41	10702.63	10629.30
42	10708.29	10766.12
43	10736.40	10769.63
44	10789.13	10841.46
45	10794.99	10854.75
46	10801.06	11503.43
47	10816.70	11541.28
48	10862.64	11560.51
49	10889.36	11560.85
50	10907.99	11563.97
….	….	….

Table S12. QDPT-SOC mixing arising from CASSCF/NEVPT2 states. Only the first 9 states displayed.

SOC-state energy	CASSCF/NEVPT2 State	|S, Ms >	Weight
0 cm^{-1}			
	0	|1,1>	0.303003
	1	|1,1>	0.046098
	2	|1,1>	0.018282
	0	|1,0>	0.169671
	1	|1,0>	0.014207
	2	|1,0>	0.067211
	0	|1,-1>	0.303003
	1	|1,-1>	0.046098
	2	|1,-1>	0.018282
90,18 cm^{-1}			
	0	|1,1>	0.345492
	1	|1,1>	0.076667
	0	|1,0>	0.099253
	1	|1,0>	0.043017
	0	|1,-1>	0.345492
	1	|1,-1>	0.076667
91,98 cm^{-1}			
	0	|1,1>	0.136553
	2	|1,1>	0.075613
	0	|1,0>	0.520021
	2	|1,0>	0.042271
	0	|1,-1>	0.136553
	2	|1,-1>	0.075613
658,67 cm^{-1}			
	1	|1,1>	0.104582
	2	|1,1>	0.191788
	1	|1,0>	0.291321
	2	|1,0>	0.095894
	1	|1,-1>	0.104582
	2	|1,-1>	0.191788
717,30 cm^{-1}			
	1	|1,1>	0.199917
	2	|1,1>	0.102616
	1	|1,0>	0.100012
	2	|1,0>	0.285829
	1	|1,-1>	0.199917
	2	|1,-1>	0.102616
1.047,23 cm^{-1}			
	0	|1,1>	0.085711
	1	|1,1>	0.300413
	0	|1,0>	0.027266
	1	|1,0>	0.187546
	0	|1,-1>	0.085711
	1	|1,-1>	0.300413
1.053,06 cm^{-1}			
	1	|1,1>	0.082339
	2	|1,1>	0.265642
	0	|1,0>	0.014207
	1	|1,0>	0.261596
	1	|1,-1>	0.082339
	2	|1,-1>	0.265642
1.278,83 cm^{-1}			
	0	|1,1>	0.082325
	1	|1,1>	0.165850
	2	|1,1>	0.069761
	0	|1,0>	0.046158
	1	|1,0>	0.051561
	2	|1,0>	0.254918
	0	|1,-1>	0.082325
	1	|1,-1>	0.165850
	2	|1,-1>	0.069761

Table S13. *ZFS* (*D* and *E*) and g-values obtained from different SOC operators.

SOC Method	*D*/cm^{-1} and *E*/cm^{-1}	*g*-tensor
SOCType-1 (effective nuclear charge)	*D* = 88; *E* = 0.78	g_{iso} = 2.73 (41)
SOCType-2 (mean-field with atomic densities read from disk)	*D* = 162; *E* = 1,37	g_{iso} = 2.81 (00)
SOCType-3 (mean-field/effective potential) (default)	*D* = 91; *E* = 0,9	g_{iso}= 2.74 (03)
SOMF(1X)	*D* = 90; *E* = 0,8	g_{iso}= 2.73 (86)
RI-SOMF(1X)	*D* = 90; *E* = 0,8	g_{iso}= 2.73 (86)
VEFF-SOC	*D* = 110; *E* = 0,9	g_{iso}= 2.76 (89)
VEFF(-2X)-SOC	*D* = 91; *E* = 0,8	g_{iso}= 2.74 (12)
AMFI	*D* = 90; *E* = 0,8	g_{iso} = 2.73 (90)
CASSCF/NEVPT2 18e – 14o	*D* = 95; *E* = 2	g_{iso}= 2.71 (27)

6. References

[1] E. W. Bastiaan, MacLean, C., Zijl, P.C.M.v. & Bothner-By, A.A. , *Ann. Rep. NMR Spect.* **1987**, *19*, 35-77.

[2] G. A. Bain, J. F. Berry, *J. Chem. Educ.* **2008**, *85*, 532.

[3] K. Kabsch, in *International Tables for Crystallography* (Eds.: M. G. Rossmann, E. Arnold), Kluwer Academic Publishers, Dordrecht, The Netherlands, **2001**.

[4] *SAINT*, Bruker AXS GmbH, Karlsruhe, Germany, **1997-2013**

[5] *CrysAlis, PRO*, Rigaku Oxford Diffraction, Wroclaw, Poland, **2015 - 2025**

[6] R. Blessing, *Acta Cryst. A* **1995**, *51*, 33-38.

[7] *SADABS*, Bruker AXS GmbH, Karlsruhe, Germany, **2004-2014**

[8] *SCALE3 ABSPACK, CrysAlisPro*, Rigaku Oxford Diffraction, Rigaku Polska Sp.z o.o., Wrocław, Poland, **2015-2025**

[9] W. R. Busing, H. A. Levy, *Acta Cryst.* **1957**, *10*, 180-182.

[10] O. V. Dolomanov, L. J. Bourhis, R. J. Gildea, J. A. Howard, H. Puschmann, *Appl. Cryst.* **2009**, *42*, 339-341.

[11] *SHELXL-20xx*, University of Göttingen and Bruker AXS GmbH, Karlsruhe, Germany, **2012-2018**

[12] G. Sheldrick, *Acta Cryst. C* **2015**, *C71*, 3-8.

[13] G. Sheldrick, *Acta Cryst. A* **2008**, *A64*, 112-122.

[14] G. Sheldrick, *Acta Cryst. C* **2015**, *71*, 3-8.

[15] P. Müller, R. Herbst-Irmer, A. Spek, T. Schneider, M. Sawaya, Oxford: Oxford Science Publications, **2006**.

[16] D. Watkin, *Appl. Cryst.* **2008**, *41*, 491-522.

[17] S. K. Singh, J. Eng, M. Atanasov, F. Neese, *Coord. Chem. Rev.* **2017**, *344*, 2-25.
